# Use of Sedation During Non-Invasive Ventilation in Intensive Care Unit: A Systematic Review and Meta-Analysis

**DOI:** 10.3390/jpm16070385

**Published:** 2026-07-19

**Authors:** Carmine Iacovazzo, Andrea Uriel de Siena, Katarzyna Kotfis, Pasquale Buonanno, Serena Nappi, Raffaele Merola, Maria Vargas, Giuseppe Servillo, Pratik P. Pandharipande, Annachiara Marra

**Affiliations:** 1 Department of Neurosciences, Reproductive and Odontostomatological Sciences, University of Naples, Federico II, 80131 Naples, Italy; iacovazzo@tin.it (C.I.); andreauriel@outlook.it (A.U.d.S.); pasquale.buonanno@unina.it (P.B.); vargas.maria82@gmail.com (M.V.);; 2 Department of Anesthesiology, Intensive Care and Pain Management, Pomeranian Medical University in Szczecin, 70-110 Szczecin, Poland; 3 Division of Anesthesiology Critical Care Medicine, Department of Anesthesiology, Vanderbilt University Medical Center, Nashville, TN 37232, USA; pratik.pandharipande@vumc.org; 4 Critical Illness, Brain Dysfunction, and Survivorship Center, Vanderbilt University Medical Center, Nashville, TN 37203, USA; 5 Division of Pulmonary and Critical Care Medicine, Department of Medicine, Vanderbilt University Medical Center, Nashville, TN 37232, USA

**Keywords:** acute respiratory failure, non-invasive ventilation, personalized sedation, precision medicine, meta-analysis

## Abstract

**Background**: Non-invasive ventilation (NIV) is a well-established approach for preventing endotracheal intubation (ETI) in critically ill patients with acute respiratory failure (ARF). Sedation is frequently used to improve comfort. This study aimed to analyze the impact of sedation during NIV on ETI rates and NIV success in critically ill patients. **Methods**: We systematically searched in PubMed, EMBASE, MEDLINE, Web of Science, and CENTRAL up to September 2023, including prospective observational studies, retrospective cohort studies (nRCTs), and randomized controlled trials (RCTs). Primary outcomes were NIV success and ETI rates; secondary outcomes were hypotension, bradycardia, 28-day mortality, delirium, and oversedation. Proportions were used for observational studies, odds ratios (OR) for retrospective studies, and risk ratios (RR) for RCTs. Retrospective studies compared intermittent and continuous analgosedation, while RCTs evaluated dexmedetomidine versus other sedatives, including direct comparisons. **Results**: Four observational studies, 2 retrospective studies, and 7 RCTs (738 patients) were selected. Dexmedetomidine showed increased NIV success (RR = 1.167, 95%C.I. 1.014–1.343, *p* = 0.032) and reduced ETI rate (RR = 0.553, 95%C.I. 0.405–0.755, *p* < 0.001), but higher rate of bradycardia (RR = 2.172, 95%C.I. 1.819–4.042, *p* < 0.001) and hypotension (RR = 2.441, 95%C.I. 1.608–3.706, *p* < 0.001). nRCTs revealed higher NIV success (Proportion = 0.694, 95%C.I. 0.528–0.912, *p* = 0.009), moderate ETI rates (Proportion = 0.379, 95%C.I. 0.282–0.511, *p* < 0.001), and low bradycardia and hypotension rates. **Conclusions**: Our findings suggest that sedation, particularly dexmedetomidine-based strategies, may enhance NIV success and lower ETI rates. However, dexmedetomidine was also associated with higher rates of bradycardia and hypotension, especially compared with midazolam. To establish the correct sedation strategy, it is important to tailor the drug to the patient, considering its hemodynamic instability, delirium risk, and mortality risk.

## 1. Introduction

Over the last few decades, non-invasive ventilation (NIV) has been increasingly used in patients with acute respiratory failure (ARF) to prevent or avoid endotracheal intubation (EIT), especially in those with chronic obstructive pulmonary disease (COPD), pneumonia, cardiogenic pulmonary edema (COeARF), and more recently, acute respiratory distress syndrome (ARDS) [1,2,3]. There is mounting evidence that the use of NIV in ARF in intensive care units (ICUs) has positive outcomes on length of stay, pneumonia frequency, ETI rate, and mortality [4,5,6]. Furthermore, the shortage of ICU beds, especially during the COVID-19 pandemic, growing confidence in the technique, and the opportunity to treat hypercapnic alone or mild hypoxemic ARF in its earlier stages led to NIV application outside of the ICU [2].

NIV success depends significantly on the patient’s tolerance and coordination with ventilation. Factors such as pain, pressure sores, agitation, stress, discomfort, or claustrophobia can lead to low tolerance and acceptance of NIV [7,8]. Additionally, NIV may be influenced by the patient–device interface and associated air leaks, the severity of the disease condition, agitation, and the mode and settings of NIV being used. These factors may necessitate the judicious use of sedation during NIV treatment to enhance patient tolerance [9,10]. However, sedation can have dangerous side effects, including respiratory depression, bradycardia, hypotension, delirium, and increased mortality [11]. The decision to use sedatives and/or analgesics during NIV therapy remains controversial, as there is a lack of clear guidelines. Considering this background, it is important to analyze the current evidence and take into account all patient’s risk factors to establish the best tailored strategy for critically ill patients.

The aim of this systematic review and meta-analysis is to evaluate the efficacy and safety of sedation for critically ill patients with ARF receiving NIV in the ICU.

## 2. Materials and Methods

### 2.1. Search Strategy

This systematic review and meta-analysis followed a protocol published in the Prospective Register for Systematic Reviews (CRD42023396360) and was conducted according to the guidelines in the Preferred Reporting Items for Systematic Reviews and Meta-analyses (PRISMA); the PRISMA checklist is reported in Table S1 (Supplemental Materials) [12].

We conducted a comprehensive search for all observational, prospective, and randomized controlled trials (RCTs) evaluating the use of sedation during NIV in adult patients with ARF admitted to the ICU. The search was performed in the following electronic databases up to September 2023: Cochrane Central Register of Controlled Trials (CENTRAL), Embase, MEDLINE, PubMed, and Web of Science. No language restrictions were applied. Table S2 (Supplemental Materials) reports the search strategies.

### 2.2. Inclusion and Exclusion Criteria

Eligible studies reported at least one of the following outcomes: NIV success rate (NIV failure was defined as the need for ETI within the first 72 h after NIV starting; accordingly, NIV success was defined as avoidance of ETI during this period); mortality at 28 days; need for ETI; hyperactive delirium (defined as RASS > 0 and positive CAM-ICU) at any time during the ICU stay; hypotension (defined as mean arterial pressure < 65 mm Hg),and bradycardia (defined as a heart rate [HR]< 60 beats/min), considered as cardiovascular complications; and oversedation (defined as a RASS score < 0).

The study population included adults (age > 18 years) admitted to the ICU with ARF, AHRF, ARDS, or COeARF, AE-COPD (Acute Exacerbation of Chronic obstructive pulmonary disease), and PORF (Post-Operative Respiratory Failure) treated with NIV associated with the use of sedation. We analyzed the following agents: alpha2-agonists (dexmedetomidine and clonidine), hypnotic drugs (benzodiazepines and propofol), analgesic drugs (remifentanil and morphine), and other sedatives (haloperidol).

We excluded studies involving pediatric populations and patients with ARF due to chest trauma.

### 2.3. Data Extraction and Quality Assessment

Two reviewers (SN and AUdS) independently screened studies for inclusion, retrieved potentially relevant studies, and decided on study eligibility using a standardized data extraction form, checked by the other authors. Any disagreement was solved by discussion or by the judgment of a third author (CI). We collected the following data from every included study: authors, year of publication, study design, patient’s demographic, interventions, and results. Two investigators (CI and AC) independently screened the citations to identify other potentially eligible studies not included in the previous search. Two pairs of reviewers (RM, MV and PB, KK) independently assessed the methodological quality of eligible studies. Any disagreement was resolved through discussion or adjudication by a third author (AM and PPP). Data extraction was performed with a standardized electronic data sheet. To evaluate the overall risk of bias for RCTs, we used the Revised Cochrane Risk of Bias tool (RoB2) [13]. We evaluated five domains: randomization process, deviations from intended interventions (effect of assignment to intervention), missing outcome data, measurement of the outcome, and selection of reported results. Every domain was evaluated as ‘low’, ‘some concerns’, or ‘high’ and an overall risk of bias was assigned. To assess the risk of bias for observational prospective studies, we used the Risk of Bias In Non-randomized Studies-of Interventions (ROBINS-I) [14]. The ROBINS-I evaluates five domains: bias due to confounding; bias in selection of participants into the study; bias in classification of interventions; bias due to deviations from intended interventions; bias due to missing data, bias in measurement of outcomes; and bias in selection of the reported results. To assess the risk of bias for observational retrospective studies, we used the Risk Of Bias In Non-randomized Studies-of Exposure (ROBINS-E) [15]. We assessed seven domains: bias due to confounding; bias arising from measurement of the exposure; bias in selection of participants into the study; bias due to post-exposure interventions; bias due to missing data; bias arising from measurement of the outcome; and bias in selection of the reported results. Every domain of ROBINS-I and ROBINS-E was evaluated as ‘low’, ‘moderate’ or ‘some concerns’, ‘serious’, ‘critical’, or ‘no information’, and an overall risk of bias was assigned. We analyzed the risk of bias with the ‘robvis’ (version 0.3.1, 2019) package in R (version 4.5.1, 2025) [16,17].

### 2.4. Outcome Measures

The primary outcomes were NIV success rate and need of ETI. Secondary outcomes included mortality at 28 days, incidence of hypotension, bradycardia, oversedation, and incidence of delirium.

### 2.5. Statistical Analysis

We reported all available data for all studies. Continuous variables are presented as means and standard deviation (SD), and categorical variables are presented as number of observations and relative frequencies. We used relative risk (RR) for two-arm studies and for RCTs, odds ratio (OR) to analyze the retrospective studies, and proportions for one-arm studies. We analyzed studies with three or more arms as separated sub-studies: each intervention was compared with all the others, for each outcome. We conducted three sub-analyses for each outcome, sorted by study type, intervention, and a sensitivity analysis by excluding studies with ‘high’ or ‘very high’ risk of bias, evaluated with the above-mentioned tools. Heterogeneity was assessed by I^2^ calculation, and it was considered low, moderate, or high if I2 values were under 25%, between 25% and 50%, or over 50%, respectively [15]. All results were presented as Forrest plots. We considered a *p*-value < 0.05 as significant. All analyses were conducted with ‘R’ (version 4.5.1, 2025), using ‘metafor’ (version 4.6-0, 2024) package [17,18].

### 2.6. Fragility Index and Trial Sequential Analysis

We evaluated the robustness of each trial by calculating the fragility indices (FI) of each RCT for each outcome. For every outcome, the median, interquartile range (IQR), lowest, and maximum were determined for each FI. All calculations were performed using R (version 4.5.1, 2025) [17,19].

To construct a Trial Sequential Analysis (TSA) graph, we plotted the cumulative z-score against the cumulative number of participants included across studies for each outcome [20]. On the *x*-axis, the cumulative number of participants enrolled across studies is displayed chronologically, while the *y*-axis shows the cumulative z-score, reflecting the statistical strength of the accumulated data as each study is added. Horizontal red lines at z-scores of −1.96 and 1.96 represent statistical significance thresholds, corresponding to a *p*-value of 0.05, indicating that if the cumulative z-score crosses these lines, the result has reached conventional significance [20]. The TSA graph includes sloping red lines, referred to as benefit and harm boundaries, which act as monitoring boundaries. These boundaries are designed to assess significance for either benefit or harm: when the cumulative z-score line (often displayed in blue) crosses one of these boundaries, the result can be considered statistically significant. A vertical red line, representing the required information size (RIS), denotes the sample size needed to reliably detect or rule out the assumed effect size; this value is adjusted based on study heterogeneity. The RIS was estimated using relative risk reduction and heterogeneity-adjusted information size for dichotomous outcomes [20]. If the cumulative z-score crosses this RIS line, the outcome can be considered conclusive for the selected effect. Finally, diverging purple lines from the *x*-axis represent futility boundaries. When the cumulative z-score crosses into this futility area, it suggests that even with additional studies, a statistically significant difference between groups is unlikely, signaling limited utility in further study accumulation [20].

## 3. Results

**Figure 1:**
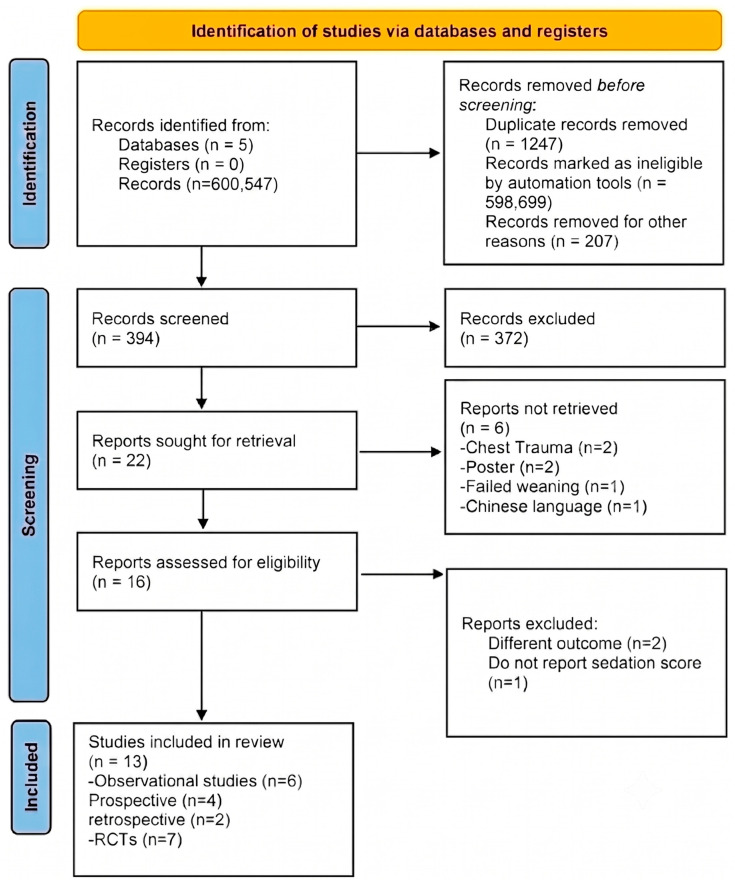
PRISMA Flowchart.

After screening, 22 reports were retrieved and 6 studies were excluded for the following reasons: 1 article about chest trauma patients, 2 posters, 1 article about patients with failed weaning, 1 for different outcomes, and 1 for the absence of sedation scores. After the first screening, 16 studies were assessed for eligibility and 3 were excluded: 2 because of different outcomes and 1 because of a missing sedation score; finally, a total of 13 studies were included in the systematic review [21,22,23,24,25,26,27,28,29,30,31,32,33,34,35] (Figure 1). We included 7 randomized controlled trials (6 two-arm studies [21,22,23,25,26,27], 1 three-arm study [24]), and 6 observational studies (4 prospective one-arm studies [30,31,32,33], 2 retrospective studies [28,29]). The total number of patients of the selected studies was 738 (553 patients from RCTs, 116 from prospective studies, and 69 from retrospective studies). Haloperidol and remifentanil were administered in only one study; we were therefore unable to perform a subgroup analysis for this drug. Table S3 (Supplementary Materials, SM) summarizes the characteristics of the enrolled and analyzed studies. All the studies used a sedation scale to guide the sedation: 2 articles used the RASS scale with a target -2/1 [21,22]; one with a RASS -2/1 [23]; 4 studies with a RASS score less than 3 [24,26,27,30]; 3 with a target of Richer sedation agitation scale (RSAS) of 2–4 [25,27,33]; one study with an Observer assessment of agitation and sedation of 3 or 4 [31]; 2 with a RASS target of -2/0 [28,29].

### 3.1. Risk of Bias Assessment

Figure S1 (Supplementary Materials, SM) reports the risk of bias assessment. According to the RoB2 score, three studies had a ‘low’ overall risk of bias [25,26,27] and three studies were judged to have ‘some concerns’ regarding overall risk of bias due to randomization process or deviations from the intended interventions. Only one study was rated as having a ‘high’ risk of bias due to ‘some concerns’ in deviations from intended intervention and missing data, and high risk in selection of the reported result [23]. According to the ROBINS-E, one study was judged as having moderate risk of bias due to confounding variables [28]. According to the ROBINS-I, only one study reported a high risk of bias due to ‘serious risk of bias’ due to confounding variables [31], and one reported an overall ‘moderate risk’ due to ‘no information’ for confounding variables and ‘some concerns’ in reporting results [35].

### 3.2. Analysis of RCTs

**Figure 2:**
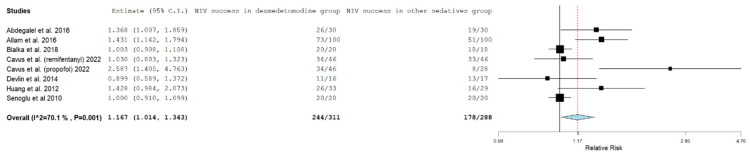
Non-invasive ventilation (NIV) success rates in RCTs [21,22,23,24,25,26,27]. The squares represent the sample size of each study, the horizontal lines represent the confidence intervals of each study, and the diamond represent the final result of the analysis.

**Figure 3:**
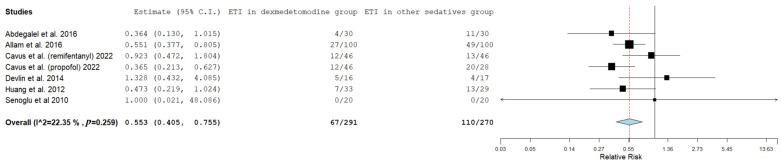
Endotracheal intubation (ETI) rates in RCTs [21,22,24,25,26,27]. The squares represent the sample size of each study, the horizontal lines represent the confidence intervals of each study, and the diamond represent the final result of the analysis.

**Figure 4:**
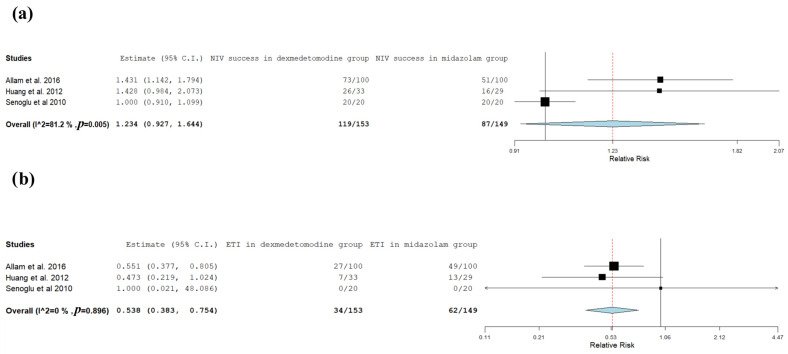
Non-invasive ventilation (NIV) and endotracheal intubation (ETI) success rates comparing dexmedetomidine and midazolam. (**a**) NIV success rate [22,26,27]; (**b**) ETI success rate [22,26,27]. The squares represent the sample size of each study, the horizontal lines represent the confidence intervals of each study, and the diamond represent the final result of the analysis.

**Figure 5:**
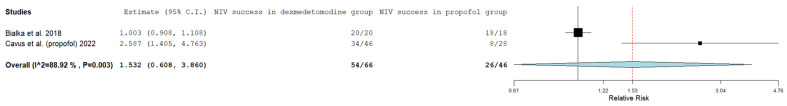
Non-invasive ventilation (NIV) success rate comparing dexmedetomidine and propofol [23,24]. The squares represent the sample size of each study, the horizontal lines represent the confidence intervals of each study, and the diamond represent the final result of the analysis.

Figure 2 and Figure 3 report the analysis of NIV success and ETI rates among the RCTs, respectively. The use of dexmedetomidine was associated with a higher NIV success rate compared to the other sedatives (RR = 1.167, 95%C.I. 1.014–1.343, I^2^ = 70.1%, *p* = 0.032) and a lower rate of ETI (RR = 0.553, 95%C.I. 0.405–0.755, I^2^ = 22.35%, *p* < 0.001); the sub-analysis shows that dexmedetomidine had a lower risk of ETI compared to midazolam (Figure 4b) (RR = 0.538, 95%C.I. 0.383–0.754, I^2^ = 0%, *p* = 0.896). No differences were found between dexmedetomidine and midazolam (Figure 4a) or propofol (Figure 5) in NIV success rate. No other comparisons could be performed due to lack of data.

Figure S2 (SM) reports the sensitivity analysis of the NIV success rate and shows an increase in NIV success rate in the dexmedetomidine group (RR = 1.234, 95%C.I. 1.019–1.496, I^2^ = 72.23%, *p* = 0.002), with a moderate heterogeneity. The TSA of NIV success and ETI rate are reported in Figure S3 (SM). Only the analyses of ETI rates between dexmedetomidine and other sedatives (Figure S3b, *n* = 320) and midazolam (Figure S3d, *n* = 187) reached the RIS and confirmed the lower ETI rate in the dexmedetomidine group. No other analyses, and neither NIV success rate sensitive analysis (Figure S4), reached the RIS.

Figure S5 reports the bradycardia and hypotension rates and their sub-analysis. The incidences of bradycardia (Figure S5a, RR = 2.172, 95%C.I. 1.819–4.042, I^2^ = 0%, *p* < 0.001) and hypotension (Figure S5b, RR = 2.441, 95%C.I. 1.608–3.706, I^2^ = 0%, *p* < 0.001) were higher with dexmedetomidine infusion compared to the other sedatives; the comparison of bradycardia (Figure S5c, RR = 2.609, 95%C.I. 1.699–4.006, I^2^ = 0%, *p* < 0.001) rate and hypotension (Figure S5d, RR = 2.746, 95%C.I. 1.753–4.301, I^2^ = 0%, *p* < 0.001) rate between dexmedetomidine and midazolam were higher in the dexmedetomidine group. No differences were found in bradycardia (Figure S5e) and hypotension (Figure S5f) rates between dexmedetomidine and propofol. No other sub-analysis could be performed due to lack of data. These results support the concept of precision sedation during NIV, in which the choice of sedative agent should be guided by the expected clinical benefit and the individual patient’s risk profile, especially hemodynamic vulnerability and delirium risk.

Figure S6 (SM) report the sensitivity analysis of bradycardia and hypotension: it confirms the risks for higher bradycardia rate (Figure S6a, RR = 2.744, 95%C.I. 1.837–4.099, I^2^ = 0%, *p* < 0.001) and hypotension rate (Figure S6b, RR = 2.441, 95%C.I. 1.608–3.706, I^2^ = 0%, *p* < 0.001) in the dexmedetomidine group. Figure S7 (SM) reports the TSA of bradycardia and hypotension comparisons. The analyses of bradycardia reached the RIS in the general (Figure S7a, *n* = 162) and midazolam (*n* = 104) sub-analyses and in the sensitivity analysis (Figure S7c, *n* = 150), confirming a higher risk in the dexmedetomidine group, as with the hypotension events (Figure S7b, *n* = 267; Figure S7d, *n* = 119). The propofol sub-analysis did not reach the RIS. The sensitivity analysis (Figure S8) reached the required RIS. Therefore, while dexmedetomidine was associated with improved NIV-related outcomes, its increased risk of bradycardia and hypotension suggests that sedative selection should be individualized according to baseline cardiovascular status and the need for close monitoring.

Figure S9 shows the comparisons of delirium, 28-day mortality, and oversedation rates. No differences were found between the dexmedetomidine and the other sedatives; no difference was found between the two drugs in risk of delirium with moderate heterogeneity (I^2^ = 67.72%) between dexmedetomidine and midazolam; moreover, dexmedetomidine showed a reduction in the risk of mortality (Figure S9d, RR = 0.27, 95%C.I. 0.073–0.994, I^2^ = 24.9%, *p* = 0.049). No sensitivity analysis was conducted due to lack of data. Figure S10 (SM) shows the TSA for delirium, 28-day mortality, and oversedation respectively. Although none of the analyses reached the RIS, the analysis of 28-day mortality (Figure S10b,c) suggests a possible risk of delirium with the use of the other sedatives.

### 3.3. Analysis of Non-Randomized Trials

Figure S11 shows the comparisons between intermittent strategy and continuous sedation strategy in retrospective studies and the results from observational studies. No differences were found between the groups for ETI and 28-day mortality (Figure S11a,b). Figure S12 (SM) shows the TSA of retrospective studies which did not achieve the RIS. The NIV success did not appear to be clearly associated with the use of a sedative (Figure S11c, proportion = 0.727, 95%C.I. 0.547–0.965, I^2^ = 75.59%, *p* = 0.084), with a moderate link with ETI success (Figure S11d, proportion = 0.349, 95%C.I. 0.249–0.49, I^2^ = 0%, *p* < 0.001); moreover, sedation does not seem to influence the bradycardia (Figure S11e, proportion = 0.042, 95%C.I. 0.009–0.201, I^2^ = 0%, *p* < 0.001) and hypotension (Figure S11f, proportion = 0.032, 95%C.I. 0.008–0.124, I^2^ = 0%, *p* < 0.001). The 28-day mortality was relatively linked with the administration of sedatives (Figure S11g, proportion = 0.141, 95%C.I. 0.151–0.398, I^2^ = 0%, *p* < 0.001), as was oversedation (Figure S11h, proportion = 0.078, 95%C.I. 0.017–0.360, I^2^ = 0%, *p* = 0.001).

Figure S13 (SM) shows the sensitivity analysis of observational studies. The sensitivity analysis of NIV success and ETI rate showed a possible link between success with sedation (Figure S13a, proportion = 0.754, 95%C.I. 0.548–1.038, I^2^ = 80.92%, *p* = 0.028), (Figure S13b, proportion = 0.339, 95%C.I. 0.206–0.558, I^2^ = 20.65%, *p* < 0.001), and a similar result for bradycardia (Figure S13c, proportion = 0.040, 95%C.I. 0.006–0.275, I^2^ = 0%, *p* < 0.001) and hypotension (Figure S13d, proportion = 0.028, 95%C.I. 0.006–0.136, I^2^ = 0%, *p* < 0.001). The sensitivity analysis of mortality showed a possible link with a low mortality at 28 days with sedation (Figure S13e, proportion = 0.164, 95%C.I. 0.056–0.478, I^2^ = 36.47%, *p* < 0.001). These results highlight the importance of sedation use in high-risk patients to avoid ETI.

### 3.4. Fragility Index Analysis

Table S4 reports the fragility indexes (FI) and its descriptive analysis. The descriptive analysis showed that the median of FI for all outcomes was very low (Median = 0); only one study reported a FI > 8 for more than one outcome. Generally, the robustness of the studies was very low for all outcomes.

## 4. Discussion

This systematic review and meta-analysis aimed to assess the efficacy and safety of sedation for critically ill patients with ARF receiving NIV in the ICU. In our analysis, dexmedetomidine was associated with a higher incidence of bradycardia and hypotension compared with midazolam, while showing a comparable risk profile to propofol, although the strength of this evidence remains limited. These findings are clinically relevant because hemodynamic adverse events may offset the potential benefits of improved NIV tolerance, particularly in patients with shock, cardiac dysfunction, conduction abnormalities, or pre-existing hemodynamic instability. Therefore, dexmedetomidine should not be considered universally preferable; rather, its use should be guided by individual patient characteristics, baseline cardiovascular status, and the possibility of close hemodynamic monitoring. Table S5 summarizes the difference between our manuscript and previous studies [34,35,36]. Sedation may facilitate patient–ventilator synchrony and improve interface tolerability; however, interpretation of these findings is constrained by the limited robustness of the available evidence. These proposed mechanisms, including improved comfort, better patient–ventilator synchrony, and reduced respiratory drive, may partly explain the higher NIV success observed with dexmedetomidine or other sedative agents. Pooled analysis suggests a potential association between sedation and higher NIV success and lower ETI rates. However, it is important to underline that no drug is without adverse events and it is mandatory to tailor the sedation strategy to patients, also considering analgesia strategy, before starting the sedative [37]. Therefore, evidence supporting these findings primarily derives from a dexmedetomidine-based trial, and the robustness of these findings was limited, with a median fragility index of 0, and no consistent differences across sedative agents. Patient–ventilator asynchrony during NIV is a well-recognized contributor to discomfort and treatment failure, driven by mismatches between patient inspiratory effort and ventilator support, exacerbated by leaks and increased work of breathing [38]. Excessive respiratory drive and anxiety may further worsen trigger delay, double triggering, or ineffective efforts. Judiciously titrated sedation may attenuate sympathetic activation and excessive inspiratory effort, improve comfort with the interface, and promote more stable patient–ventilator interaction. These physiological mechanisms may partly explain the higher NIV success observed with dexmedetomidine or other sedative agents, although the available evidence remains heterogeneous and does not establish causality. This issue has recently led to the proposal of the concept of lung-protective sedation [39]: using this sedation, it is possible to reduce patient self-induced lung injury (P-SILI) and ventilator-induced lung injury (VILI) by avoiding ineffective effort or giant tidal volume. According to other studies, desaturation and discomfort are common causes of tracheal intubation in various patient populations [39,40,41]. Improved patient adaptation to the NIV interface likely enhances oxygenation and reduces episodes of desaturation, thereby decreasing the need for tracheal intubation. In our analysis, no significant differences in ETI rates were observed when comparing dexmedetomidine with midazolam or propofol, suggesting a comparable effect across commonly used sedative agents. Nonetheless, propofol, although frequently used, may increase patient–ventilator asynchrony by promoting upper airway collapsibility and reducing arousal, and has been associated with higher rates of delirium [41,42]. Therefore, its use during NIV requires careful titration and close monitoring [38,42]. These findings underscore the importance of considering patient comfort and the careful selection of sedative agents to optimize NIV outcomes in critically ill patients.

The use of sedative agents was shown to be likely associated with a reduction in 28-day mortality in critically ill patients treated with NIV as derived from RCTs and double-arm nRCTs analyses [34]: our findings suggest no significant differences between dexmedetomidine, midazolam, and propofol. It is important to underline that these findings are not strong; more studies are needed to establish the differences between the analyzed drugs. This reduction in mortality can be attributed to the avoidance of tracheal intubation, based on the TSA of RCTs. Tracheal intubation carries the risk of several complications, including ventilator-associated pneumonia, tracheomalacia, laryngeal injury, vocal cord paralysis, tube displacement, tracheobronchitis, fistulas between the trachea and adjacent structures, and tracheal tube shuttering [34]. These complications can be life-threatening and significantly increase the 28-day mortality rate. By improving NIV tolerance, analgosedation may help avoid tracheal intubation and its associated complications. However, this potential benefit must be balanced against adverse events, particularly bradycardia, for which TSA reached the RIS. This, in turn, enhances patient outcomes by reducing the incidence of these potentially severe complications, thereby contributing to lower mortality rates.

By improving tolerance to NIV, analgosedation could potentially reduce the need for tracheal intubation and, consequently, limit exposure to these complications. Overall, these findings highlight the potential role of sedation in improving patient comfort and patient–ventilator synchrony during NIV. Nevertheless, any potential improvement in NIV tolerance should be weighed against the increased risk of bradycardia and hypotension observed with dexmedetomidine, particularly when compared with midazolam. Moreover, given the limited robustness of the evidence, further well-designed studies that also consider patient-reported outcome measures, are needed to clarify whether different sedation strategies influence clinically relevant outcomes and to identify the most appropriate sedative agent.

In our analysis, dexmedetomidine was associated with a higher incidence of bradycardia and hypotension compared with midazolam, while showing a comparable risk profile to propofol, as suggested by RCTs analysis, although the strength of this evidence remains limited. One possible reason for these side effects is the pharmacodynamics of these drugs. Dexmedetomidine is an alpha2-agonist, and hypotension can be explained by two mechanisms: a direct mechanism involving pre-synaptic and post-synaptic alpha2-receptors, and an indirect mechanism due to bradycardia and the consequent reduction in cardiac output [43]. Midazolam and propofol, on the other hand, are GABAergic drugs that activate the GABA receptor and inhibit the sympathetic nervous system, which can also induce hypotension [44]. Understanding these pharmacodynamic properties is crucial for selecting the most appropriate sedative drug for individual patients, particularly those with pre-existing cardiovascular conditions. Indeed, this approach is more adherent to a precision medicine model. Proper selection and management of sedative agents can help mitigate the risk of adverse cardiovascular events, thereby enhancing the overall safety and efficacy of sedation in critically ill patients undergoing NIV.

We observed that the rates of delirium and oversedation seems to be lower in the dexmedetomidine group compared to other sedatives’ groups, with no significant difference reported between midazolam and placebo, considering that no analysis reached the RIS. According to other studies, oversedation is a risk factor for delirium, and dexmedetomidine is commonly less associated with delirium in critically ill patients. However, in our study, no significant difference in delirium rates was observed between dexmedetomidine and midazolam, largely due to the limited availability of data on delirium incidence. Therefore, choosing a sedative that minimizes the risk of delirium is crucial for improving patient outcomes and the use of tools to predict the risk of delirium could be safer for the patient. Our study emphasizes the need for further research to fully understand the impact of different sedatives on delirium rates in critically ill patients considering that no analysis reached the RIS to fully establish the safest sedative agents for hemodynamic stability. Collecting more comprehensive data on delirium incidence in future studies will help clarify the comparative benefits of dexmedetomidine and midazolam, enabling clinicians to make more informed and tailored decisions about sedation management in the ICU.

Our findings should be interpreted in the context of several limitations. It was not possible to make more detailed comparisons between dexmedetomidine and each sedative due to insufficient data. Additionally, there was a lack of evidence regarding the use of haloperidol and other sedatives, reducing the generalizability of these results. Second, heterogeneity in NIV interfaces and variability in the definitions and timing of NIV success across included studies may have influenced the pooled estimates. Moreover, several analyses were affected by small sample sizes and heterogeneity, and the overall robustness of the evidence was low, as reflected by very low fragility indices and trial sequential analyses that did not reach the required information size [45]. In addition, pooled proportions derived from one-arm prospective studies may be influenced by confounding by indication and selection bias. Finally, GRADE methodology was not applied. This represents an important limitation because the certainty of evidence is likely low or very low for several key outcomes, including NIV success, endotracheal intubation, bradycardia, hypotension, delirium, and mortality. This judgment is supported by the heterogeneity of study designs, limited sample sizes, variable risk of bias, low fragility indices, and the fact that several trial sequential analyses did not reach the required amount of information. Therefore, these findings should be interpreted as hypothesis-generating rather than definitive evidence for clinical recommendations. To mitigate the impact of these limitations, we restricted comparisons to studies with similar designs, conducted a rigorous assessment of risk of bias, and systematically evaluated the robustness of the findings. Nevertheless, these methodological constraints warrant cautious interpretation of the results. Despite these efforts, the limitations still highlight the need for caution when interpreting our results. Future research should focus on addressing these limitations by including a broader range of sedatives, standardizing the types of interfaces used, and improving study design and robustness. This will help provide clearer insights into the efficacy and safety of sedation in critically ill patients receiving NIV in the ICU.

## 5. Conclusions

In conclusion, sedation during NIV appears to be associated with an increased likelihood of NIV success and a reduced need for endotracheal intubation in critically ill patients, based on evidence derived from RCT analysis. Midazolam, propofol, and dexmedetomidine showed a possible relationship with increased NIV success and fewer endotracheal intubation events, as suggested by single-arm nRCT analysis. However, causality cannot be established based on the available evidence for haloperidol, midazolam, and propofol; only for dexmedetomidine could a causality link be hypothesized, considering the population of our study. Although more studies are needed to clarify the role of sedation during NIV, current evidence suggests that sedation is a valuable tool to enhance NIV success with a relatively low rate of adverse events. Drug choice should be tailored to the individual patient, with particular attention to delirium, hemodynamic adverse events, and 28-day mortality. Therefore, while sedation may represent a potentially useful adjunct during NIV, its use should be individualized according to patient characteristics, risk factors and clinical context. Drug choice should be tailored to the individual patient, with particular attention to delirium, hemodynamic adverse events, and 28-day mortality, considering its morbidities and characteristics. Although dexmedetomidine may improve NIV tolerance and reduce ETI rates, its association with increased bradycardia and hypotension requires careful patient selection and close hemodynamic monitoring, especially in patients with shock, cardiac dysfunction, conduction abnormalities, or hemodynamic instability. Therefore, these potential benefits should be balanced against hemodynamic adverse events, and sedative choice should be individualized towards patients with careful monitoring. Because the GRADE methodology was not applied, and given the heterogeneity of study designs, limited sample size, variable risk of bias, and low fragility indices, the certainty of evidence should be considered low or very low for several outcomes. Further well-designed studies are needed to better define the optimal sedation strategy and to strengthen the evidence base.

## Data Availability

All the data were collected by the included article. An electronic dataset is available to the corresponding author upon reasonable request.

## Supplementary Materials

The following supporting information can be downloaded at: https://www.mdpi.com/article/10.3390/jpm16070385/s1. Table S1. PRISMA Checklist; Table S2. Search queries for each screened databases; Table S3: Study characteristics and population; Table S4: The fragility indexes of each study and outcome with median and interquartile range (IQR), minimum and maximum; Table S5: Comparison between the present study and other similar articles published previously; Figure S1: Risk of bias assessment with Cochrane tools: traffic light plot on the left, summary plot on the right; Figure S2: Sensitivity analysis of non-invasive ventilation (NIV) success rate comparison between dexmedetomidine and other sedatives; Figure S3: Trial sequential analysis (TSA) of non-invasive ventilation (NIV) and endotracheal intubation (ETI) comparisons in randomized controlled trials: (a,b) TSA of NIV success and ETI rate comparisons, respectively, between dexmedetomidine and other sedatives; (c,d) TSA of NIV success and ETI rate comparisons, respectively, between dexmedetomidine and midazolam; (e) TSA of NIV success rate comparison between dexmedetomidine and propofol; Figure S4: Trial sequential analysis (TSA) of sensitivity analysis of non-invasive ventilation; Figure S5: Trial sequential analysis (TSA) of bradycardia and hypotension rates in randomized controlled trials: (a,b) TSA of bradycardia and hypotension rate comparisons, respectively, between dexmedetomidine and other sedatives; (c,d) TSA of bradycardia and hypotension rate comparisons, respectively, between dexmedetomidine and midazolam; (e,f) TSA of bradycardia and hypotension rate comparisons, respectively, between dexmedetomidine and propofol; Figure S6: Sensitivity analysis of bradycardia and hypotension between dexmedetomidine and other sedatives: (a) bradycardia comparison; (b) hypotension comparison; Figure S7: Trial sequential analysis (TSA) of analysis of bradycardia and hypotension rates in randomized controlled trials: (a,b) TSA of bradycardia and hypotension rates comparisons, respectively, between dexmedetomidine and other sedative; (c,d) TSA of bradycardia and hypotension rates comparisons, respectively, between dexmedetomidine and midazolam; (e,f) TSA of bradycardia and hypotension rates comparisons, respectively, between dexmedetomidine and propofol; Figure S8: Trial sequential analysis (TSA) of sensitivity comparison of bradycardia and hypotension between dexmedetomidine and other sedatives: (a) bradycardia; (b) hypotension; Figure S9: Delirium rates, 28-day mortality, and oversedation events in randomized controlled trials: (a,b) delirium and 28-day mortality rates comparisons, respectively, between dexmedetomidine and other sedatives; (c,d) delirium and 28-day mortality rates comparisons, respectively, between dexmedetomidine and midazolam; (e) oversedation rates comparison between dexmedetomidine and other sedatives; Figure S10: Trial sequential analysis (TSA) of delirium, 28-day mortality, and oversedation rates in randomized controlled trials: (a,b) TSA of delirium and 28-day mortality rates comparisons, respectively, between dexmedetomidine and other sedatives; (c,d) TSA of delirium and 28-day mortality rates comparisons, respectively, between dexmedetomidine and midazolam; (e) TSA of oversedation rate comparison between dexmedetomidine and other sedatives; Figure S11: Results from non-randomized clinical trials: (a) endotracheal intubation rate (ETI) comparison between dexmedetomidine and other sedatives in retrospective studies; (b) 28-day mortality rate comparison between dexmedetomidine and other sedatives in retrospective studies; (c) NIV success rate in observational studies; (d) ETI rate in observational studies; (e) bradycardia events in observational studies; (f) hypotension events in observational studies; (g) 28-day mortality event in observational studies; (h) oversedation rate in observational studies; Figure S12: Trial sequential analysis (TSA) of retrospective studies: (a) TSA of endotracheal intubation (ETI) rates comparison between dexmedetomidine and other sedatives; (b) TSA of 30 days mortality rate comparison between dexmedetomidine and other sedatives; Figure S13: Results from sensitivity analysis of observational studies: (a) non-invasive ventilation (NIV) success proportion in sedation group; (b) endotracheal intubation (ETI) rate in sedation group; (c) bradycardia events in sedation group; (d) hypotension events in sedation group; (e) 28-day mortality rate in sedation group.

## Abbreviations

ACPE	Acute Cardiogenic Pulmonary Edema
ACRF	Acute Cardiogenic Respiratory Failure
AE-COPD	Acute Exacerbation of Chronic obstructive Pulmonary Disease
AHRF	Acute hypoxemic respiratory failure
ALI	Acute lung injury
APACHE	Acute Physiology and Chronic Health Evaluation
ARDS	Acute respiratory distress syndrome
ARF	Acute respiratory failure
BP	Blood Pressure
CI	Confidence Interval
CoeARF	Cardiogenic edema acute respiratory failure
COPD	Chronic-obstructive pulmonary disease
ETI	Endotracheal intubation
FI	Frailty Indexes
FiO2	Fraction inspired of oxygen
IQR	Interquartile range
GCS	Glasgow Coma Scale
ICU	Intensive care unit
log	Logarithmic
LOS	Length of stay
NIMV	Non-Invasive mechanical ventilation
NIV	Non-invasive ventilation
OAA/S	Observer assessment of alert/sedation scale
OR	Odds Ratio
P	*p*-value
PaO2	Arterial pressure of oxygen
PORF	Post-Operative Respiratory Failure
PRISMA	Preferred Reporting Items for Systematic Reviews and Meta-Analyses
RASS	Richmond agitation-sedation scale
RCTs	randomized controlled trials
ROB-2	Revised Cochrane Risk-of-Bias tool
RR	Risk Ratio
SD	Standard deviation
SM	Supplementary Materials
SpO2	Peripheral saturation of oxygen
TCI	Target controlled infusion
