# Supplementary material for: Use of Sedation During Non-Invasive Ventilation in Intensive Care Unit: A Systematic Review and Meta-Analysis
Source: J Pers Med. 2026 Jul 19;16(7):385. doi: 10.3390/jpm16070385 (PMC13412897; doi:10.3390/jpm16070385)

Table S1. PRISMA Checklist.

Section and Topic	Item #	Checklist item	Location where item is reported
**TITLE**			
Title	1	Identify the report as a systematic review.	Title
**ABSTRACT**			
Abstract	2	See the PRISMA 2020 for Abstracts checklist.	Abstract
**INTRODUCTION**			
Rationale	3	Describe the rationale for the review in the context of existing knowledge.	Introduction
Objectives	4	Provide an explicit statement of the objective(s) or question(s) the review addresses.	Introductionfinal part
**METHODS**			
Eligibility criteria	5	Specify the inclusion and exclusion criteria for the review and how studies were grouped for the syntheses.	Methods- Inclusion and exclusion criteria
Information sources	6	Specify all databases, registers, websites, organisations, reference lists and other sources searched or consulted to identify studies. Specify the date when each source was last searched or consulted.	Methods – Search strategy
Search strategy	7	Present the full search strategies for all databases, registers and websites, including any filters and limits used.	Methods – Search strategy Appendix 1 – Supplementary Materials
Selection process	8	Specify the methods used to decide whether a study met the inclusion criteria of the review, including how many reviewers screened each record and each report retrieved, whether they worked independently, and if applicable, details of automation tools used in the process.	Methods – Search strategy

Section and Topic	Item #	Checklist item	Location where item is reported
Data collection process	9	Specify the methods used to collect data from reports, including how many reviewers collected data from each report, whether they worked independently, any processes for obtaining or confirming data from study investigators, and if applicable, details of automation tools used in the process.	Methods – Data extraction and quality assessment
Data items	10a	List and define all outcomes for which data were sought. Specify whether all results that were compatible with each outcome domain in each study were sought (e.g. for all measures, time points, analyses), and if not, the methods used to decide which results to collect.	Methods- Outcome measures
	10b	List and define all other variables for which data were sought (e.g. participant and intervention characteristics, funding sources). Describe any assumptions made about any missing or unclear information.	Methods- Outcome measures
Study risk of bias assessment	11	Specify the methods used to assess risk of bias in the included studies, including details of the tool(s) used, how many reviewers assessed each study and whether they worked independently, and if applicable, details of automation tools used in the process.	Methods – Data extraction and quality assessment
Effect measures	12	Specify for each outcome the effect measure(s) (e.g. risk ratio, mean difference) used in the synthesis or presentation of results.	Methods- Outcome measures
Synthesis methods	13a	Describe the processes used to decide which studies were eligible for each synthesis (e.g. tabulating the study intervention characteristics and comparing against the planned groups for each synthesis (item #5)).	Methods
	13b	Describe any methods required to prepare the data for presentation or synthesis, such as handling of missing summary statistics, or data conversions.	Methods- Statistical analysis
	13c	Describe any methods used to tabulate or visually display results of individual studies and syntheses.	Methods- Statistical analysis
	13d	Describe any methods used to synthesize results and provide a rationale for the choice(s). If meta-analysis was performed, describe the model(s), method(s) to identify the presence and extent of statistical heterogeneity, and software package(s) used.	Methods- Statistical analysis

	13e	Describe any methods used to explore possible causes of heterogeneity among study results (e.g. subgroup analysis, meta-regression).	Methods-Statistical analysis
	13f	Describe any sensitivity analyses conducted to assess robustness of the synthesized results.	Methods-Statistical analysis
Reporting bias assessment	14	Describe any methods used to assess risk of bias due to missing results in a synthesis (arising from reporting biases).	Methods-Statistical analysis
Certainty assessment	15	Describe any methods used to assess certainty (or confidence) in the body of evidence for an outcome.	Methods-Statistical analysis
**RESULTS**			
Study selection	16a	Describe the results of the search and selection process, from the number of records identified in the search to the number of studies included in the review, ideally using a flow diagram.	Results Figure 1
	16b	Cite studies that might appear to meet the inclusion criteria, but which were excluded, and explain why they were excluded.	Results
Study characteristics	17	Cite each included study and present its characteristics.	Results
Risk of bias in studies	18	Present assessments of risk of bias for each included study.	Results Figure 1S
Results of individual studies	19	For all outcomes, present, for each study: (a) summary statistics for each group (where appropriate) and (b) an effect estimate and its precision (e.g. confidence/credible interval), ideally using structured tables or plots.	Results Figures 2, 3, 4, 5,2S,3S, 4S, 5S, 6S, 7S, 8S

**Section and Topic**	**Item #**	**Checklist item**	**Location where item is reported**
Results of syntheses	20a	For each synthesis, briefly summarise the characteristics and risk of bias among contributing studies.	Results Discussion

	20b	Present results of all statistical syntheses conducted. If meta-analysis was done, present for each the summary estimate and its precision (e.g.<br>confidence/credible interval) and measures of statistical heterogeneity. If comparing groups, describe the direction of the effect.	Results<br>Figures 2, 3, 4, 5,2S,3S, 4S, 5S,<br>6S, 7S, 8S
	20c	Present results of all investigations of possible causes of heterogeneity among study results.	Results<br>Figures 2, 3, 4, 5,2S,3S, 4S, 5S,<br>6S, 7S, 8S
	20d	Present results of all sensitivity analyses conducted to assess the robustness of the synthesized results.	Results<br>Figures 2, 3, 4, 5,2S,3S, 4S, 5S,<br>6S, 7S, 8S
Reporting biases	21	Present assessments of risk of bias due to missing results (arising from reporting biases) for each synthesis assessed.	Results<br>Figure 1S
Certainty of evidence	22	Present assessments of certainty (or confidence) in the body of evidence for each outcome assessed.	Results<br>Figures 2, 3, 4, 5,2S,3S, 4S, 5S,<br>6S, 7S, 8S
**DISCUSSION**			
Discussion	23a	Provide a general interpretation of the results in the context of other evidence.	Discussion
	23b	Discuss any limitations of the evidence included in the review.	Discussion
	23c	Discuss any limitations of the review processes used.	Discussion
	23d	Discuss implications of the results for practice, policy, and future research.	Discussion
**OTHER INFORMATION**			
Registration and protocol	24a	Provide registration information for the review, including register name and registration number, or state that the review was not registered.	Methods
	24b	Indicate where the review protocol can be accessed, or state that a protocol was not prepared.	Methods
	24c	Describe and explain any amendments to information provided at registration or in the protocol.	Methods
Support	25	Describe sources of financial or non-financial support for the review, and the role of the funders or sponsors in the review.	Declarations

Competing interests	26	Declare any competing interests of review authors.	Declarations
Availability of data, code and other materials	27	Report which of the following are publicly available and where they can be found: template data collection forms; data extracted from included studies; data used for all analyses; analytic code; any other materials used in the review.	Appendix 1 Declarations

From: Page MJ, McKenzie JE, Bossuyt PM, Boutron I, Hoffmann TC, Mulrow CD, et al. The PRISMA 2020 statement: an updated guideline for reporting systematic reviews. BMJ 2021;372:n71. doi: 10.1136/bmj.n71. This work is licensed under CC BY 4.0. To view a copy of this license, visit https://creativecommons.org/licenses/by/4.0/

Table S2. Search queries for each screened databases.

Database	*Search query*
Pubmed	("Noninvasive Ventilation"[MeSH Terms] OR ("Noninvasive Ventilation"[MeSH Terms] OR ("noninvasive"[All Fields] AND "ventilation"[All Fields]) OR "Noninvasive Ventilation"[All Fields] OR ("non"[All Fields] AND "invasive"[All Fields] AND "ventilation"[All Fields]) OR "non invasive ventilation"[All Fields]) OR "NIV"[All Fields] OR ("Noninvasive Ventilation"[MeSH Terms] OR ("noninvasive"[All Fields] AND "ventilation"[All Fields]) OR "Noninvasive Ventilation"[All Fields] OR ("non"[All Fields] AND "invasive"[All Fields] AND "ventilation"[All Fields]) OR "non invasive ventilation"[All Fields])) AND ("hypnotics and sedatives/administration and dosage"[MeSH Terms] OR "hypnotics and sedatives/pharmacology"[MeSH Terms] OR "hypnotics and sedatives/therapeutic use"[MeSH Terms] OR ("sedate"[All Fields] OR "sedated"[All Fields] OR "sedating"[All Fields] OR "sedation"[All Fields] OR "sedations"[All Fields]) OR ("hypnotics and sedatives"[Pharmacological Action] OR "hypnotics and sedatives"[MeSH Terms] OR ("hypnotics"[All Fields] AND "sedatives"[All Fields]) OR "hypnotics and sedatives"[All Fields] OR "sedative"[All Fields] OR "sedatives"[All Fields]) OR ("hypnosis"[MeSH Terms] OR "hypnosis"[All Fields] OR "hypnotism"[All Fields] OR "hypnotically"[All Fields] OR "hypnotics and sedatives"[Pharmacological Action] OR "hypnotics and sedatives"[MeSH Terms] OR ("hypnotics"[All Fields] AND "sedatives"[All Fields]) OR "hypnotics and sedatives"[All Fields] OR "hypnotic"[All Fields] OR "hypnotics"[All Fields] OR "hypnotized"[All Fields])) AND ("Intensive Care Units"[MeSH Terms] OR "Critical Illness"[MeSH Terms] OR (("Critical Illness"[MeSH Terms] OR ("critical"[All Fields] AND "illness"[All Fields]) OR "Critical Illness"[All Fields] OR ("critically"[All Fields] AND "ill"[All Fields]) OR "critically ill"[All Fields]) AND ("patient s"[All Fields] OR "patients"[MeSH Terms] OR "patients"[All Fields] OR "patient"[All Fields] OR "patients s"[All Fields])) OR ("Intensive Care Units"[MeSH Terms] OR ("intensive"[All Fields] AND "care"[All Fields] AND "units"[All Fields]) OR "Intensive Care Units"[All Fields] OR ("intensive"[All Fields] AND "care"[All Fields] AND "unit"[All Fields]) OR "intensive care unit"[All Fields]) OR ("Intensive Care Units"[MeSH Terms] OR ("intensive"[All Fields] AND "care"[All Fields] AND "units"[All Fields]) OR "Intensive Care Units"[All Fields] OR "icu"[All Fields]))

Medline

#	Searches	Results
1	non invasive ventilation.mp. or exp Noninvasive Ventilation/	7443
2	intensive care unit.mp. or exp Intensive Care Units/	207585
3	*Critical Illness/	21496
4	sedation.mp.	53451
5	*Analgesia/ or analgesia.mp.	93621
6	2 or 3	218325
7	4 or 5	138431
8	1 and 6 and 7	7

EMBASE

'noninvasive ventilation'/syn AND 'intensive care unit'/exp OR 'critically ill patient'/syn AND 'sedative agent'/syn

CENTRAL

ID	Search	Hits
#1	MeSH descriptor: [Noninvasive Ventilation] explode all trees	572
#2	non invasive ventilation OR NIV	5144
#3	#1 OR #2	5327
#4	MeSH descriptor: [Hypnotics and Sedatives] explode all trees	4947
#5	MeSH descriptor: [Critical Illness] this term only	3810
#6	MeSH descriptor: [Intensive Care Units] explode all trees	6283
#7	#5 OR #6	8885
#8	#3 AND #7 OR intensive care unit AND #4 OR sedatives OR sedation	30551
#9	MeSH descriptor: [Conscious Sedation] this term only	1729
#10	#3 AND #7 OR intensive care unit AND #4 OR sedatives OR #9	7726

Web of Science	1: (ALL=(NIV)) OR ALL=(Non-invasive ventilation)<br>2: (((ALL=(sedation)) OR ALL=(sedative)) OR ALL=(hypnotics)) OR ALL=(analgo-sedation)<br>3: (((ALL=(ICU)) OR ALL=(intensive care unit)) OR ALL=(critically ill patients)) OR ALL=(critical patients)<br>4: #3 AND #2 AND #1

MeSH, Medical Sub Headings

Table S3. Study characteristics and population

Study (study type)	Arms (sample size)	Administration	Study population	Definition of ARF	Dose	Inclusion criteria	Exclusion criteria	Sedation goal	Outcome	side effects	interface	Narcotics (drugs)
Abdegal et al. 2016 (RCT)	Dex (30 pts) vs Hp (30 pts) vs P. (30 pts)	bolus + C.I..	ARF or AHRF or CoeARF or PORF.	M/D	Dex loading dose of 1.0 mg/kg IV over 10 min then 0.2-0.7 mg/kg/h IV infusion. Hp loading dose of 2.5 mg IV over 10 min then continuous infusion of 0.5-2 mg/h or saline 10 ml Ivbolus followed by 2-8 mL/h.	Age more than 18 years, patients need NIV due to acute exacerbation of ARF in COPD, patients with CoeARF and PORF patients.	Refusal, patients with known allergy to any of the studied drugs, patients with known psychiatric disorders or on antipsychotic medications, patients with severe dementia, patients with heart rate 150 beats/min or systolic blood pressure 190 mmhg, patients with prolonged QTc-time (>500 ms) or history of clinically relevant ventricular arrhythmia, patients with epilepsy or parkinsonism and pregnancy	RASS=-2 to 1	Primary: incidence of delirium CAM-ICU. Secondary: ETI needing, length of ICU stay, adverse events, mortality	bradycardia, prolonged QTc interval, hypotension	full-face mask	no

Allam et al. 2016 (RCT)	Dex (100 pts) vs mdz (100 pts)	bolus + C.I.	ARDS	Signs and symptoms of ARF distress. (a) Respiratory rate more than 35/min. (b) SpO2 less than 80%. (c) Respiratory acidosis (pH < 7.2). (d) Hypoxemic (PO2 < 60 mmHg). (e) Pulse rate more than 120/min	Dex: 1µg/kg load. 0.2-0.7 µg/kg/hr maint. Mdz: 0.05 mg/kg load. 0.05-0.1 mg/kg/hr maint.	(1) Age older than 18 years. (2) Signs and symptoms of ARF distress. (a) Respiratory rate more than 35/min. (b) SpO2 less than 80%. (c) Respiratory acidosis (pH < 7.2). (d) Hypoxemic (PO2 < 60 mmHg). (e) Pulse rate more than 120/min	Severely altered conscious level (GCS < 8/15). (2) Traumatic causes of hypoxemia (severe head/chest trauma or pneumothorax). (3) Severe pneumonia diagnosed by chest radiography. (4) Arrhythmia (required cardio version) or cardiac arrest.	RASS=-2 to 1	Primary: ETI needing. Secondary: mortality rate, sedation efficiency	delirium with MDZ, brady cardia, hypot ension	mask	no
Bialka et al. 2018 (RCT)	Dex (20 pts) vs Propofol (18 pts)	C.I.	ARF post thoracic surgery	M/D	Prop: 1 to 4 µg/kg/h Dex: 0.7-2.0 µg/kg/h	ptz age of 18–70 years; American Society of Anesthesiologists (ASA) status I–III; BMI 19–30 kg m-2; and no contraindications for drugs and anaesthesia	a lack of consent; significant coagulopathy; contraindications to epidural anaesthesia or drugs used in the protocol; chronic pain and chronic pain medications intake; chest wall neoplastic	RASS -2 to -1	Primary: hemodynamic stability. Secondary: hypotension, bradycardia, delirium or agitation	none	full-face mask	Yes, (epidural, fentanyl)

						techniques used in the protocol.	invasion; visible thoracic spine deformities; previous spinal surgery; and obesity (BMI > 30 kg m-2).					
Cavus et al. 2022 (RCT)	Dex (23 pts) vs rem (23 pts) vs propofol (28 pts)	C.I.	ARDS, pneumonia	ARF acidosis [partial pressure of carbon dioxide (PCO2) ≥ 45 mmHg], a diagnosis of COPD, RR ≥ 24 per minute, and respiratory distress, with the use of auxiliary respiratory muscles	Dex 0.2–0.7 µg/kg/h by continuous intravenous infusion L (low) 0.2 µg/kg/h by continuous intravenous infusion H (High) 0.6 µg/kg/h by continuous intravenous infusion	ptz over 18 years of age, and had NIV intolerance, admission to the ICU, ARF acidosis [partial pressure of carbon dioxide (PCO2) ≥ 45 mmHg], a diagnosis of COPD, respiratory rate (RR) ≥ 24 per minute, and respiratory distress, with the use of auxiliary respiratory muscles	congestive heart failure, neurologic disease, muscular disease, treatment rejection, hepatic failure, gastrointestinal bleeding, severe hypotension [MAP < 60 mmHg], acute cardiac ischemia, and Dex, rem, and propofol allergy	RASS <3	to evaluate the effects of Dex, rem, and propofol on the clinical outcomes in NIV intolerant patients	hypotension, bradycardia, apnea, nausea, thorax rigidity, mouth dry, hypotension + bradycardia	nose-mouth mask	Yes (M/D)

					Rem 0.03–0.1 µg/kg/h by continuous intravenous infusion L (low) 0.03 µg/kg/h by continuous intravenous infusion H 0.06 µg/kg/h by continuous intravenous infusion Propofol 0.3 mg/kg/h by continuous intravenous							
Devlin et al. 2014 (RCT)	Dex (16 pts) vs P (17 pts)	C.I.	ARF	M/D	0.2 mg/kg/hr titrated by 0.1 every 30 min to	e adult patients admitted to an ICU with ARF managed	age 85 years, systolic BP 90 mm Hg, heart rate 50 beats/min, the presence of acute	RSAS from 3 to 4	Primary: tolerability of NIV. Secondary: Delirium, NIV failure, ETI	no	full-face mask or nasal mask	Yes (fentanyl)

					0.7 mg/kg/hr	with NIV for 8 h	decompensated heart failure accompanied by a cardiac ejection fraction 25%, acute alcohol withdrawal or delirium (Intensive Care Delirium Screening Checklist score 4), 28 a history of intubation and mechanical ventilation in the past month, heart block without pacemaker use, end-stage liver failure accompanied by encephalopathy, severe dementia, and treatment with clonidine or Dex in the past 30 day		needing, NIV mortality, ICU mortality.			
Huang et al. 2012 (RCT)	Dex (33 pts) vs mdz (29 pts)	C.I.	ACRF	M/D	Dex: 0.2-0.7 µg/kg/hr maint. Mdz: 0.05-0.1 mg/kg/hr	patients older than 18 years of age; signs and symptoms consistent with CoeARF; NIV failure due to	a poor respiratory state requiring immediate intubation; a clear alternative primary diagnosis such as pneumonia; severely altered	RASS <3	Primary: ETI needing. Secondary: ICU LOS, ICU mortality, NIV duration, the developmen	brady cardia, hypot ension , deliriu m, vomiti ng, gastric	helmet or fullface mask	no

						patient refusal to continue NIV because of discomfort, claustrophob ia or marked agitation.	consciousness; any patient requiring an immediate lifesaving intervention such as cardiopulmonar y resuscitation, airway control, cardioversion or inotropic support; any patient requiring thrombolysis or percutaneous coronary intervention for acute ST-segment elevation myocardial infarction.		t of complicatio n	aspirat ion, respir atory infecti on.		
Senoglu et al. 2010 (RCT)	Dex (20 pts) vs mdz (20 pts)	loading dose + C.I.	ARF for AE-COPD	M/D	D: 1 µg/kg load, 0.5 µg/kg/hr maint. M: 0.05 mg/kg load, 0.1 mg/kg/hr maint.	age >18 years and patients with ARF failure due to acute exacerbation s of COPD in our ICU who had spontaneous breathing, but who were uncooperativ e, defined as 1 on the RSS and ≥1 on the RSAS.	immediate intubation (pH < 7.15); severe hypotension (mean arterial pressure 2× the maximum reference values of liver enzymes or history of hepatic disease), renal failure (defined by the RIFLE criteria; an acronym comprising Risk, Injury, and Failure; and	RSS from 2 to 3	Primary outcome: RSS, RSAS, and BIS. Secondary: heart rate (HR), blood pressure outcomes, and ABG at each individual time point.	overse dation	face mask	no

							Loss and End-stage kidney disease), gastrointestinal hemorrhage, and psychiatric illness (including use of antidepressant medication)					
Akada et al. 2010 (Obs/p)	Dex (10 pts)	C.I.	ARF	dyspnea of sudden onset, typical findings on chest radiograph (e.g., bilateral infiltrates, perihilar bat wing appearance, Kerley B lines, etc.), or signs of ARF distress defined by hypoxemia (Pao2/Fio2 200 mm Hg)	3 µg/kg/hr over 5 min, followed by continuous infusion at a dosage range 0.2-0.7 µg/kg/hr	patients receiving NIV who were subsequently uncooperative rated as 1 on the Ramsay score and 1 or more on the RASS	ptz in poor respiratory state requiring immediate intubation, severe hemodynamic instability, hepatic failure, renal failure, digestive tract haemorrhage, or a do-not-resuscitate or do-not-intubate order	RASS < 3	primary: NIV success secondary: hemodynamic stability	no	M/D	Yes (Mo)
Clouzeau et al. 2010 (Obs/p)	TCI propofol (10 pts)	C.I.	ARF AHRF	M/D	initially 0.4 µg/mL, increments of 0.2 µg/mL	patient refusal to continue NIV sessions because of discomfort, claustrophobia or marked agitation	severely decreased consciousness (GCS score <9) not causes by hypercapnia, haemodynamic instability despite fluid challenge, use of vasoactive agents, upper digestive tract	OAA/S level of 4 or 3	Primary: ETI needing. Secondary: development of complications	oversedation	face mask	no

							haemorrhage, known allergy to propofol, inclusion in another study, weight >150 kg or <30 kg.					
Constantin et al. 2007 (Obs/p)	Rem (13 pts)	C.I.	hypoxemia or AHRF	acute hypoxemic respiratory failure, defined as a PaO2/FIO2 ratio less than 300 mmHg without left cardiac decompensation. Acute hypercapnic respiratory failure (AHRF) with pH below 7.3 and PaCO2 above 50 mmHg	0.025 µg/kg/min increasing the infusion rate by 0.025 µg/kg/min every minute to a maximum of 0.15 µg/kg/min	NIV failure due to patient refusal to continue the NIV sessions (due to discomfort), relapsing hypoxemia upon interruption of the NIV, and marked agitation	NIV failure due to impossibility of managing copious secretions, severely decreased consciousness (GCS score less than 9) not caused by hypercapnia, absence of improved gas exchange after 30 min of NIV, severe hemodynamic instability despite fluid challenge and use of vasoactive agents (systolic arterial blood pressure less than 70 mmHg), respiratory arrest, high digestive tract hemorrhage, glucose 6 phosphate dehydrogenase	RASS scale < 3	ETI needing	no	mask	Yes (rem)

							deficiency (contraindication to methylene blue), known allergy to rem or propofol, incomprehension of the study or refusal to participate, or inclusion in another research protocol					
Rocco et al. 2010 (Obs/p)	Rem (36 pts)	C.I.	AHRF	PaO2/FiO2 lower than 200 after a trial of NPPV as first-line intervention with the aim of avoiding endotracheal intubation	0.025 µg/kg/min increasing the infusion rate by 0.010 µg/kg/min every minute to a maximum of 0.12 µg/kg/min	patients refusing continue NIV for intolerance	severe hemodynamic instability such as hypotension (mean arterial pressure lower than 60 mmHg despite fluid challenge and vasoactive drugs) or rhythm disorders, severely decreased consciousness (GCS below 12), chronic obstructive pulmonary disease, age less than 18 years, and pregnant women	RSS from 2 to 3	ETI needing	armpit braces	helmet or total face mask	Yes (rem)
Matsumo et al. 2015 (Obs/r)	Dex, midazolam or propofol, Mo, risperidone, haloperidone, other	intermittent, switched to continuous, and continuous	ARDS, ALI	SpO2 was <90% despite 10 l/min via reservoir mask; 2) PaCO2	Haloperidol 2.5–5 mg by intraveno	patients over 16 years old who underwent	M/D	RASS -2 to 0	28-day mortality, failure rate of sedation	oversedation hypotension	full-face mask	Yes (Mo/fentanyl)

	intermittent: 72 ptz continuous: 11 ptz switched to continuous: 37 ptz			levels were >45 mmHg with ARF acidosis; or 3) patients had signs of respiratory distress, including a respiratory rate >24 and increased accessory respiratory muscle us	us infusion Dex 0.2 µg/kg/h by continuous intravenous infusion Midazolam 0.03 mg/kg/h by continuous intravenous infusion Propofol 0.3 mg/kg/h by continuous intravenous infusion Mo 0.02 mg/kg/h by continuous subcutaneous infusion Fentanyl 0.05–0.1 µg/kg/h by continuo	continuous NIV due to ARF and received sedatives for agitation during NIV.			between groups	delirium		

					us subcutaneous infusion							
Muriel et al. 2015 (Obs/r)	multiples drugs, most commonly mdz and Mo<br><br>analgesic or sedative: 132 ptz analgesic and sedative: 33 ptz		COPD, astma, non-COPD, ARDS, CHF, pneumonia, trauma, sepsis	M/D	M/D	more than 2h of NIV as first-line ventilatory support in ICU from the " Investigators in the third international study on Mechanical Ventilation".	NIV less than 2 hours	RASS -2 to 0	ETI needing, 28-day mortality	M/D	facial mask, nasal mask, helmet	Yes (Mo)

RASS Richmond agitation-sedation scale RCT randomized controlled trials pts patients C.I. continuous infusion ARF acute respiratory failure AHRF acute hypoxia respiratory failure CoeARF cardiogenic pulmonary oedema acute respiratory failure PORF post-operative respiratory failure M/D missing data IV intravenous Hp haloperidol COPD chronic obstructive pulmonary disease QTc-time corrected QT time ETI endotracheal intubation CAM-ICU Confusion assessment method intensive care unit ICU intensive care unit ARDS acute respiratory disease GCS Glasgow coma scale BMI body mass index RR respiratory rate MAP mean arterial pressure H high RSS Ramsey sedation scale RSAS Ricker sedation agitation scale OAA/S Observer's Assessment of Alertness/Sedation SAS sedation agitation scale Obs/p observational prospective AE-COPD Acute exacerbation-COPD Obs/r observational retrospective Mo morphine

Table S4. The fragility indexes of each study and outcome with median and interquartile range (IQR), minimum and maximum.

Study	NIV success	Endotracheal intubation	Bradycardia	Hypotension	Delirium	Mortality	Oversedation
Abdegalel et al. 2016	0	0	0	0	0	0	-
Allam et al. 2016	8	8	17	19	0	6	-
Bialka et al. 2018	0	-	0	0	-	-	-
Cavus et al. (remifentanyl) 2022	0	0	0	0	-	2	-
Cavus et al. (propofol) 2022	7	9	0	0	-	0	-
Devlin et al. 2014	0	0	0	0	0	0	1
Huang et al. 2012	0	0	0	0	0	0	-
Senoglu et al. 2010	0	0	20	0	0	0	0
**Median (IQR)**	0 (0-1)	0 (0-6)	0 (0-9)	0 (0-8)	0 (0-20)	0 (0-19)	0 (0-0)
**Minimum; Maximum**	0;8	0;9	0;17	0;19	0;20	0;6	0;1

Table S5. Comparison between the present study and other similar articles published previously.

Study	Comparisons	Inclusion and Exclusion criteria	Number of studies and patients	Statistical Analysis	Outcomes	Results	Differences
Lewis K. et al.	Dexmedetomidine vs other sedatives.	Inclusion criteria: RCT involving ICU pts aged more 18 years old with ARF undergoing NIV.<br><br>Exclusion criteria: patients with chronic disease.	12 RCTs, N=738.	Overall analysis and sensitivity analysis according to ROB.	ETI rate, delirium, ICU LOS, mortality, NIV length, pneumonia, bradycardia and hypotension.	The study found that dexmedetomidine reduced the risk of delirium (RR = 0.34) and pneumonia (RR = 0.30) while shortening ICU length of stay (MD = -2.40 day). It also lowered the need for intubation and mechanical ventilation. However, its use was associated with an increased risk of bradycardia (RR = 2.80) and hypotension (RR = 1.98). No significant difference in mortality was observed. While the findings suggest potential benefits for patients on noninvasive ventilation (NIV), the study had some risk of bias due to limitations in randomization and missing outcome data.	No TSA or sub-group analyses according dexmedetomidine vs drugs were conducted; no observational trials were included; Pneumonia, ICU LOS, and NIV length included.

Yang et al.	Mixing of drugs vs other drugs or dexmedetomidine vs other drugs.	The inclusion criteria were:) RCTs including adult patients with ARF received NIV in ICU setting treated with two groups of sedatives and analgesics or a comparison of one group of drugs with placebo; sedative and analgesic drugs can be propofol, midazolam, dexmedetomidine, remifentanil, haloperidol, etc. ; baseline information should be comparable between these two groups<br><br>Exclusion criteria: studies that subjects were patients undergoing sequential NIV after invasive mechanical ventilation were excluded; missing data.	21 RCTs, N=1384.	Overall analysis; sensitivity analysis according to ROB; subgroup analysis dexmedetomidine vs other drugs without other stratification.	ETI rate, NIV length, ICU LOS, delirium, hypotension and bradycardia.	The study found that using sedatives and analgesics during non-invasive ventilation (NIV) significantly reduced tracheal intubation rates (RR = 0.57), delirium incidence (RR = 0.372), NIV duration (-1.41 day), and ICU length of stay (-0.79 day). Compared to other sedatives, dexmedetomidine was more effective in reducing intubation rates (RR = 0.50) and delirium (RR = 0.23). However, both groups showed a higher incidence of bradycardia (RR = 1.90 in general sedatives and RR = 3.45 in dexmedetomidine). No significant differences were observed in all-cause mortality or hypotension incidence.	No TSA or sub-group analyses according dexmedetomidine vs drugs were conducted; no observational trials were included; ICU LOS ad NIV length included.

Quickfall et al.	Mixing sedatives vs inhaled sedation.	Inclusion criteria: Studies assessing one or more sedative or analgesic medications, including opioids, benzodiazepines, intravenous anesthetics, sympatholytics (dexmedetomidine), or inhaled anesthetics. Studies evaluating at least one respiratory control parameter, such as respiratory rate, tidal volume, inspiratory effort, diaphragmatic activity, airway occlusion pressure, or gas exchange (PaO2, PaCO2, SpO2, ETCO2).<br><br>Exclusion criteria: Studies that did not assess both a study medication and a respiratory control component.	51 studies (29 RCTs, 22 observational prospective studies), N=1957.	The statistical analysis grouped studies based on whether patients were spontaneously breathing or mechanically ventilated. Gas exchange measures (EtCO2, PaO2, PaCO2, SpO2) were excluded due to irrelevance. To minimize confounding, the primary analysis included only arms where a single sedative agent was administered, while multi-sedative studies were analyzed separately. Only on-treatment measurements within 72 minutes were considered, and results were summarized using percentage change from baseline. Due to study heterogeneity, direct comparisons between sedatives were abandoned, and results were instead pooled across sedative groups. Meta-	The primary outcome assessed was the ETI rate, with secondary outcomes including incidence of delirium,NIV length, ICU LOS, all-cause mortality, and adverse events .	The study found that using sedation and analgesia during NIV reduced the need for intubation, with dexmedetomidine being particularly effective. However, no significant difference was observed in all-cause mortality between sedated and non-sedated patients.	Different PICO question; different statistical analysis approach; no sub-analysis of dexmedetomidine; no TSA analysis.

				analysis used inverse-variance weighting, and random-effects meta-regression estimated pairwise differences.			

RCT randomized controlled trials NIV non-invasive ventilation ARF acute respiratory failure ICU LOS intensive care unit length of stay ETI endotracheal intubation ROB risk of bias TSA trial sequential analysis

Figure S1. Risk of bias assessment with Cochrane tools: traffic light plot on the left, summary plot on the right.

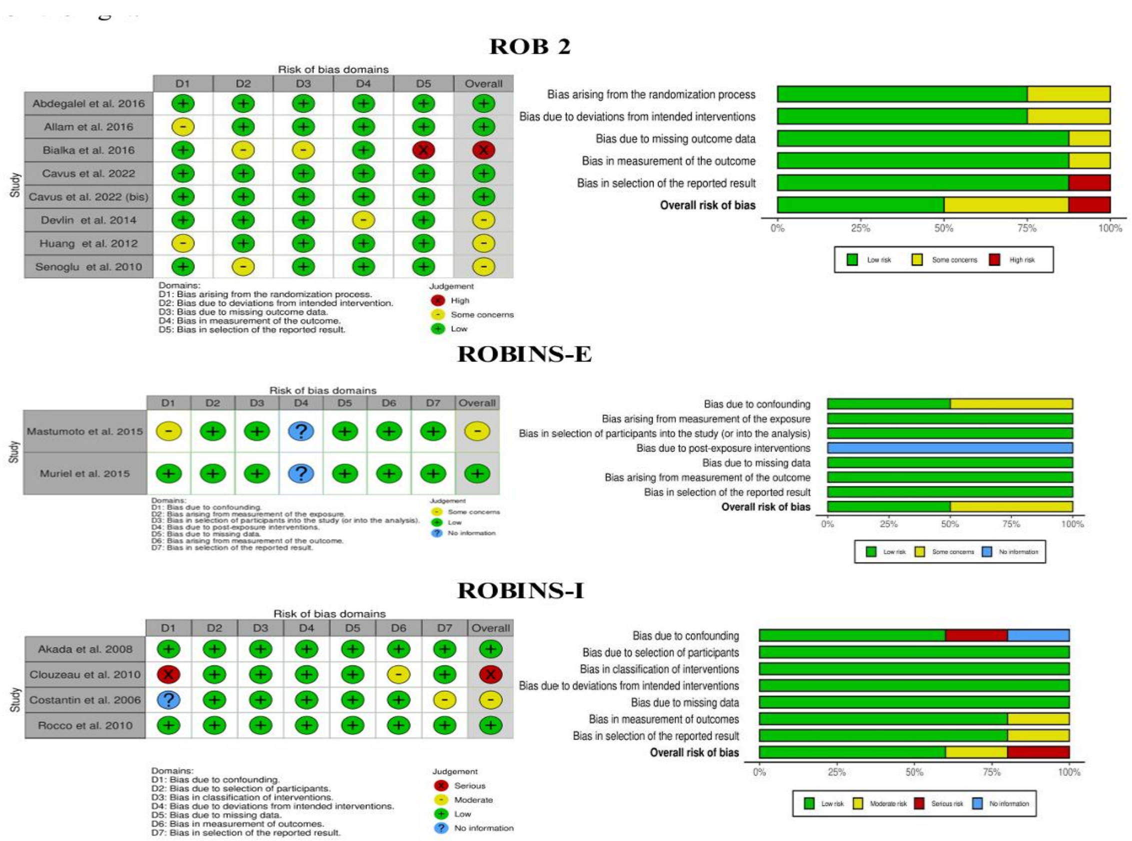

ROB 2 Risk of Bias 2; ROBINS-E Risk of Bias in non-randomized studies – of Exposure; ROBINS-I Risk of Bias in non-randomized studies – of Intervention.

Figure S2. Sensitivity analysis of non-invasive ventilation (NIV) success rate comparison between dexmedetomidine and other sedatives.

Studies	Estimate (95% C.I.)	NIV success in dexmdetomidine group	NIV success in other sedatives group
Abdegalel et al. 2016	1.368 (1.007, 1.859)	26/30	19/30
Allam et al. 2016	1.431 (1.142, 1.794)	73/100	51/100
Cavus et al. (remifentanyl) 2022	1.030 (0.803, 1.323)	34/46	33/46
Cavus et al. (propofol) 2022	2.587 (1.405, 4.763)	34/46	8/28
Devlin et al. 2014	0.899 (0.589, 1.372)	11/16	13/17
Huang et al. 2012	1.428 (0.984, 2.073)	26/33	16/29
Senoglu et al 2010	1.000 (0.910, 1.099)	20/20	20/20
**Overall (I^2=72.23 % , P=0.001)**	**1.234 (1.019, 1.496)**	**224/291**	**160/270**

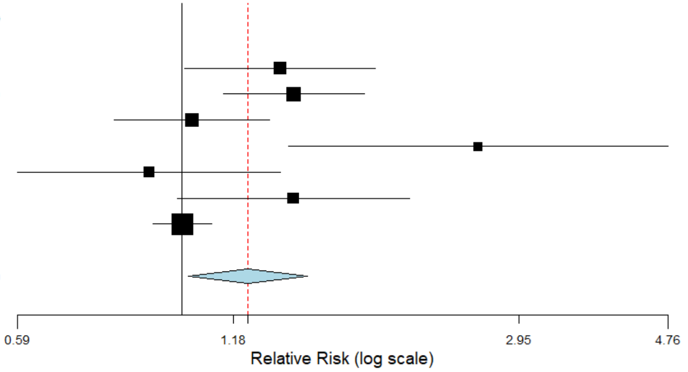

Figure S3. Trial sequential analysis (TSA) of non-invasive ventilation (NIV) and endotracheal intubation (ETI) comparisons in randomized controlled trials. a-b) TSA of NIV success and ETI rate comparisons, respectively, between dexmedetomidine and other sedatives; c-d) TSA of NIV success and EIT rates comparisons, respectively, between dexmedetomidine and midazolam; e) TSA of NIV success rate comparison between dexmedetomidine and propofol.

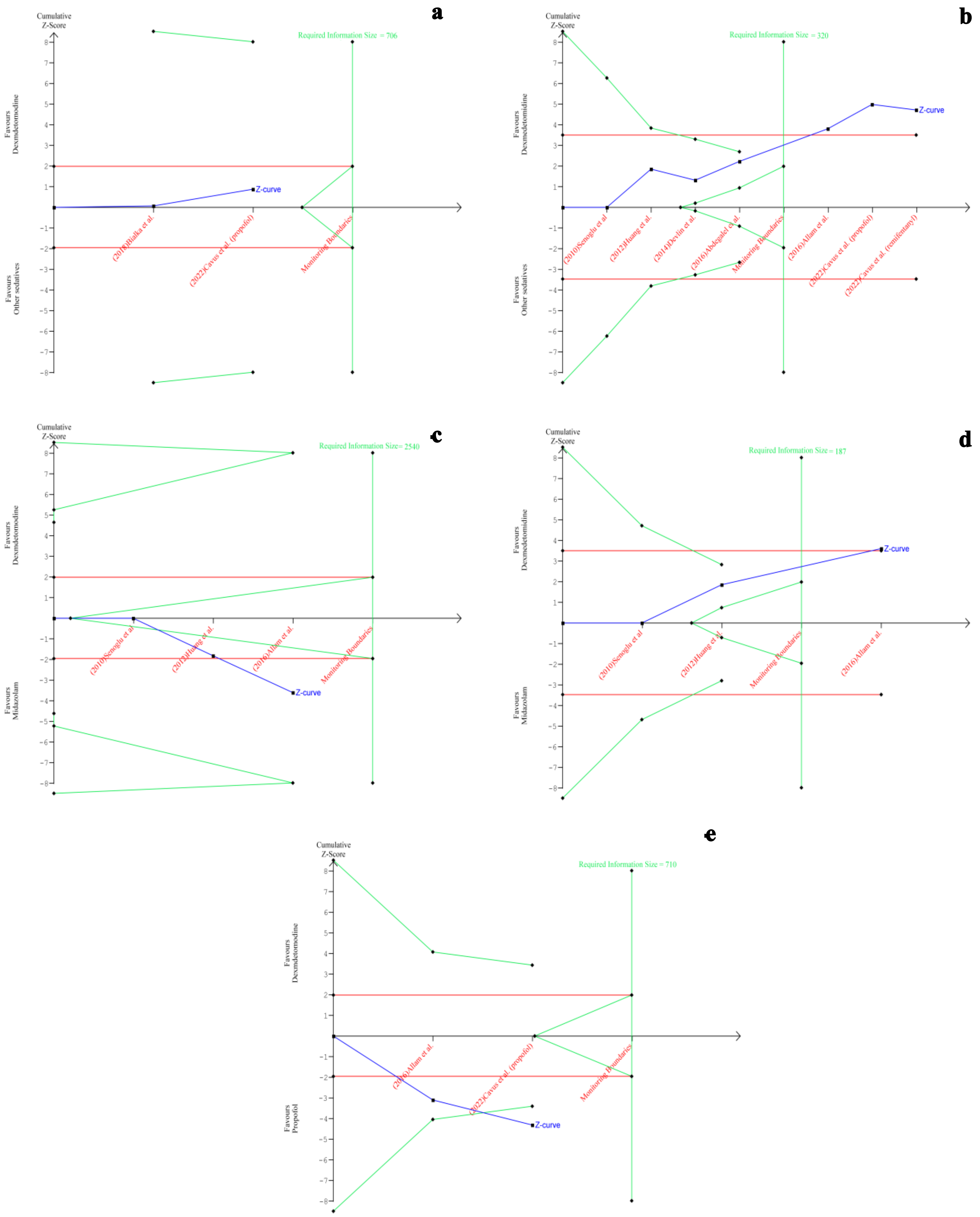

Figure S4. Trial sequential analysis (TSA) of sensitivity analysis of non-invasive ventilation.

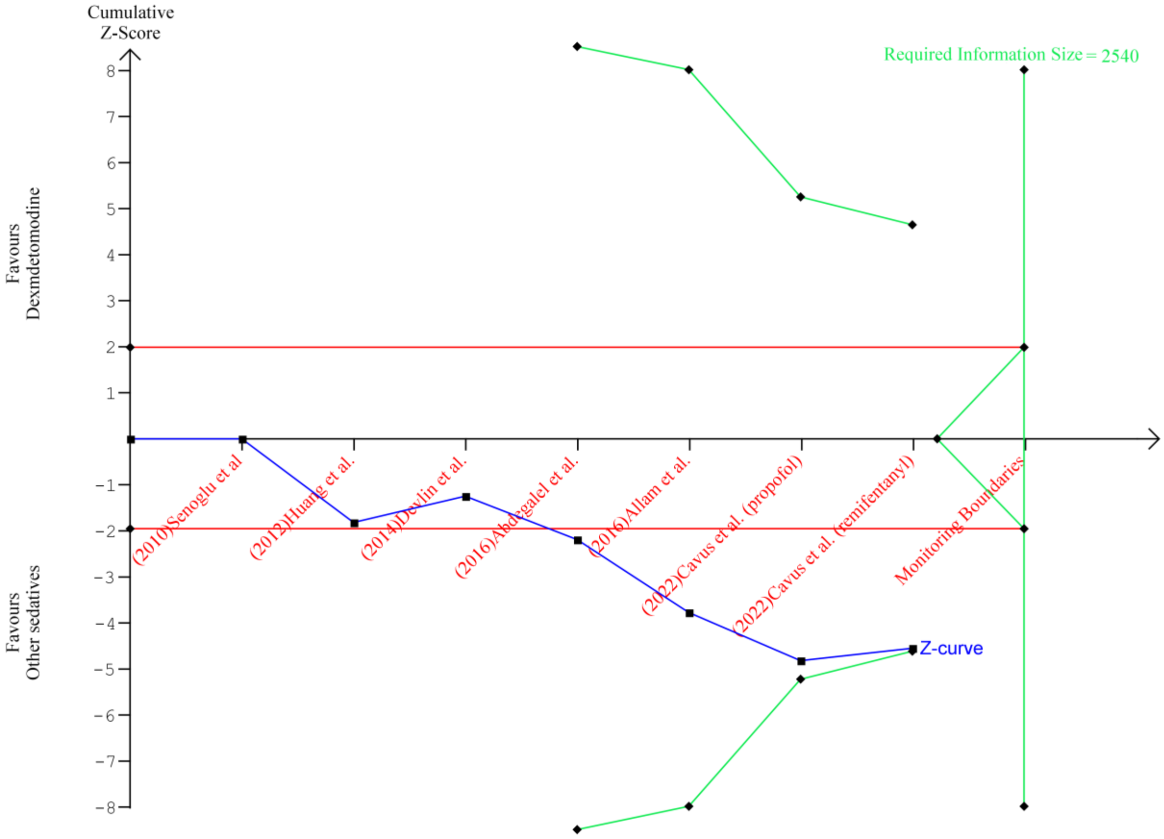

Figure S5. Bradycardia and hypotension events in randomized controlled trials. a-b) Bradycardia and hypotension events comparisons, respectively, between dexmedetomidine and other sedatives; c-d) bradycardia and hypotension events success rates comparisons, respectively, between dexmedetomidine and midazolam; e-f) bradycardia and hypotension events between dexmedetomidine and propofol.

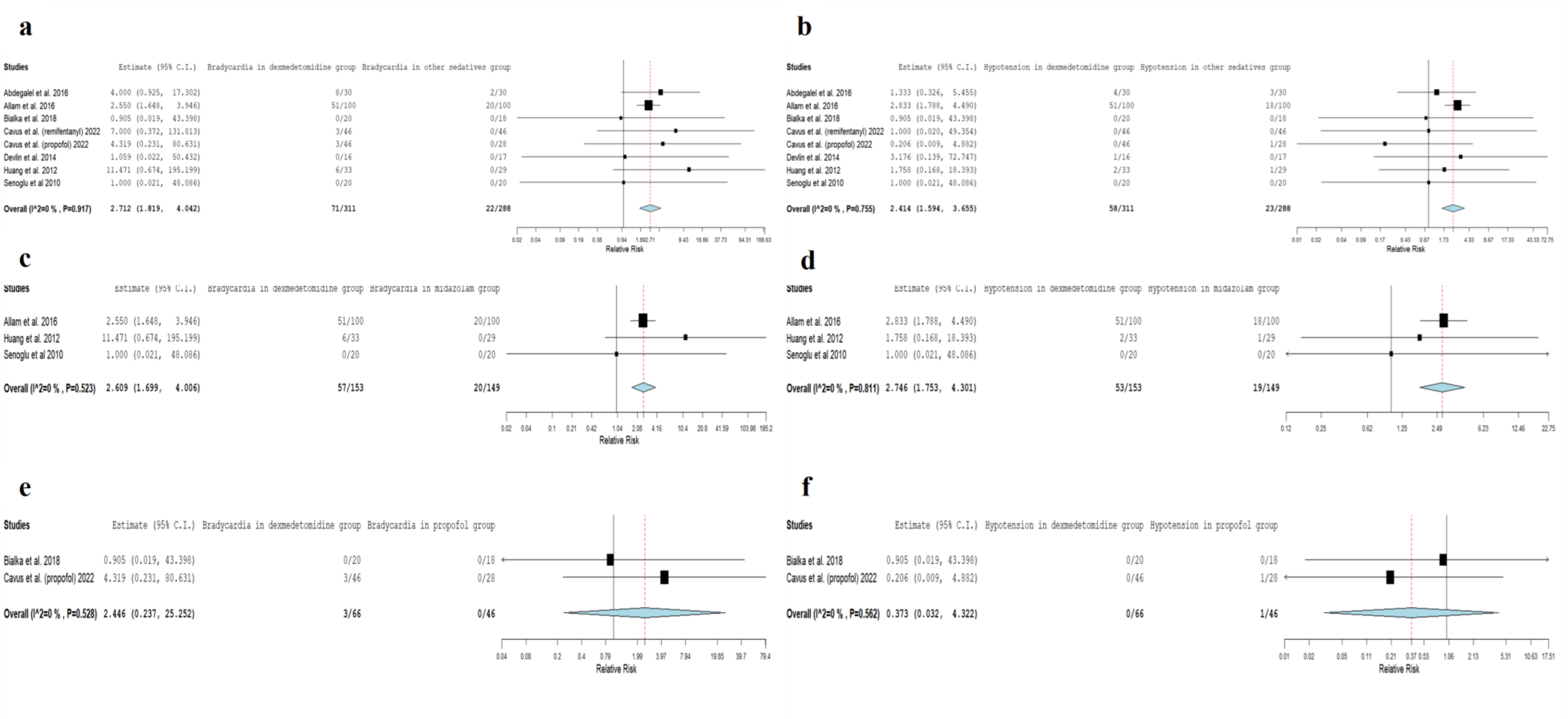

Figure S6. Sensitivity analysis of bradycardia and hypotension between dexmedetomidine and other sedatives. a) bradycardia comparison; b) hypotension comparison.

a)

Studies	Estimate (95% C.I.)	Bradycardia in dexmedetomidine group	Bradycardia in other sedatives group
Abdegalel et al. 2016	4.000 (0.925, 17.302)	8/30	2/30
Allam et al. 2016	2.550 (1.648, 3.946)	51/100	20/100
Cavus et al. (remifentanyl) 2022	7.000 (0.372, 131.813)	3/46	0/46
Cavus et al. (propofol) 2022	4.319 (0.231, 80.631)	3/46	0/28
Devlin et al. 2014	1.059 (0.022, 50.432)	0/16	0/17
Huang et al. 2012	11.471 (0.674, 195.199)	6/33	0/29
Senoglu et al 2010	1.000 (0.021, 48.086)	0/20	0/20
**Overall (I^2=0 % , P=0.888)**	**2.744 (1.837, 4.099)**	**71/291**	**22/270**

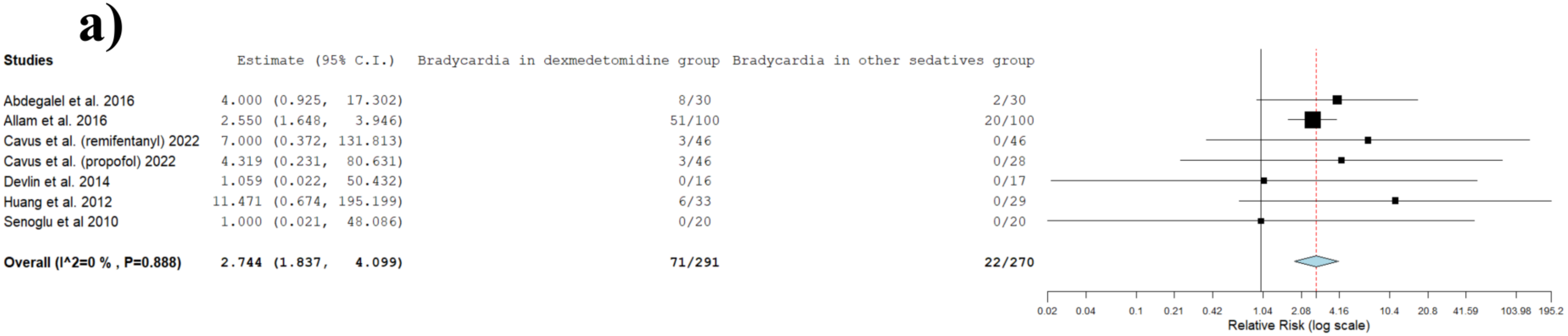

b)

Studies	Estimate (95% C.I.)	Hypotension in dexmdetomidine group	Hypotension in other sedatives group
Abdegalel et al. 2016	1.333 (0.326, 5.455)	4/30	3/30
Allam et al. 2016	2.833 (1.788, 4.490)	51/100	18/100
Cavus et al. (remifentanyl) 2022	1.000 (0.020, 49.354)	0/46	0/46
Cavus et al. (propofol) 2022	0.206 (0.009, 4.882)	0/46	1/28
Devlin et al. 2014	3.176 (0.139, 72.747)	1/16	0/17
Huang et al. 2012	1.758 (0.168, 18.393)	2/33	1/29
Senoglu et al 2010	1.000 (0.021, 48.086)	0/20	0/20
**Overall (I^2=0 % , P=0.682)**	**2.441 (1.608, 3.706)**	**58/291**	**23/270**

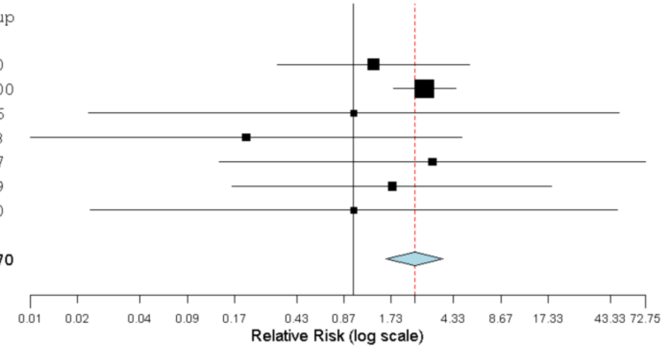

Figure S7. Trial sequential analysis (TSA) of analysis of bradycardia and hypotension rates in randomized controlled trials. a-b) TSA of bradycardia and hypotension rates comparisons, respectively, between dexmedetomidine and other sedative; c-d) TSA of bradycardia and hypotension rates comparisons, respectively, between dexmedetomidine and midazolam; e-f) TSA of bradycardia and hypotension rates comparisons, respectively, between dexmedetomidine and propofol.

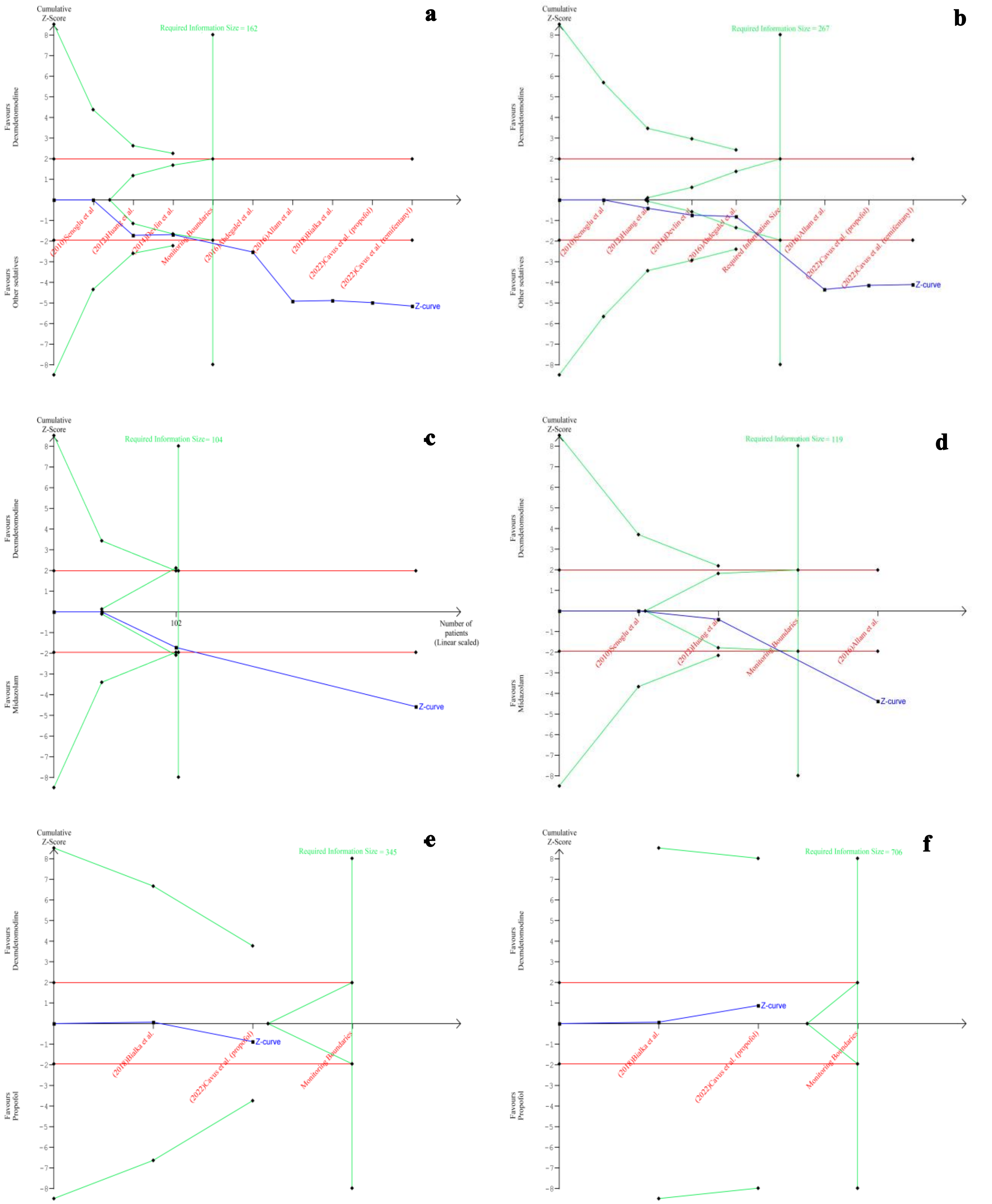

Figure S8. Trial sequential analysis (TSA) of sensitivity comparison of bradycardia and hypotension between dexmedetomidine and other sedatives. a) bradycardia; b) hypotension.

a

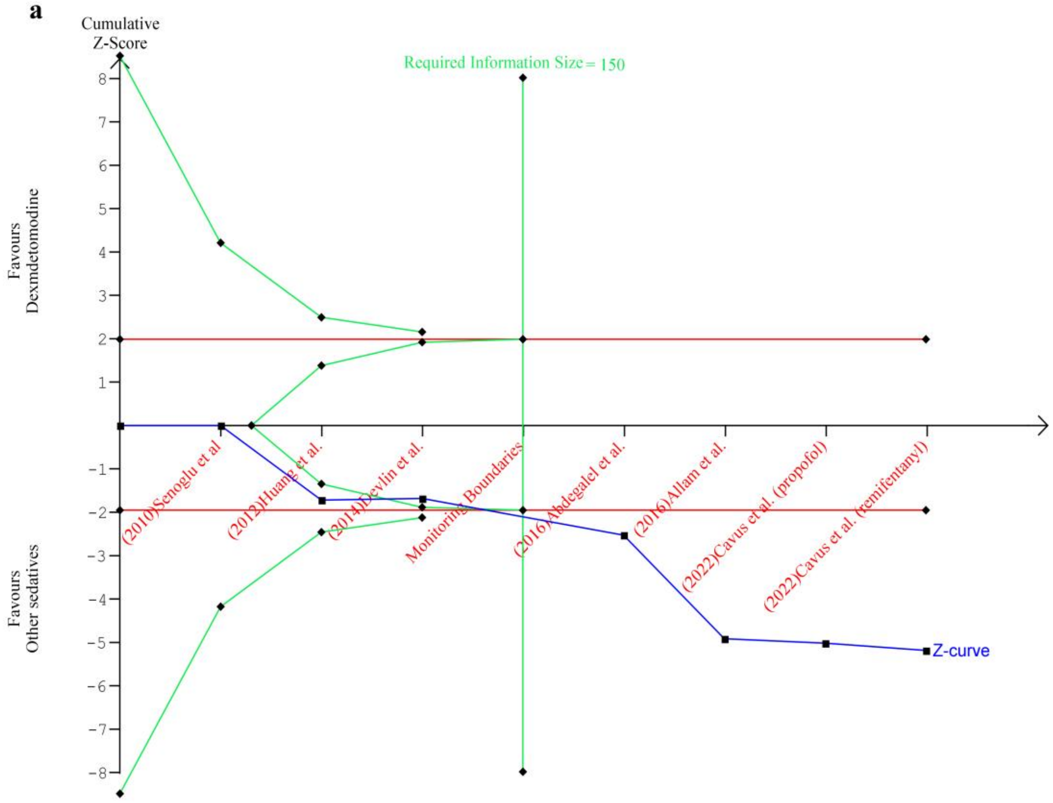

b

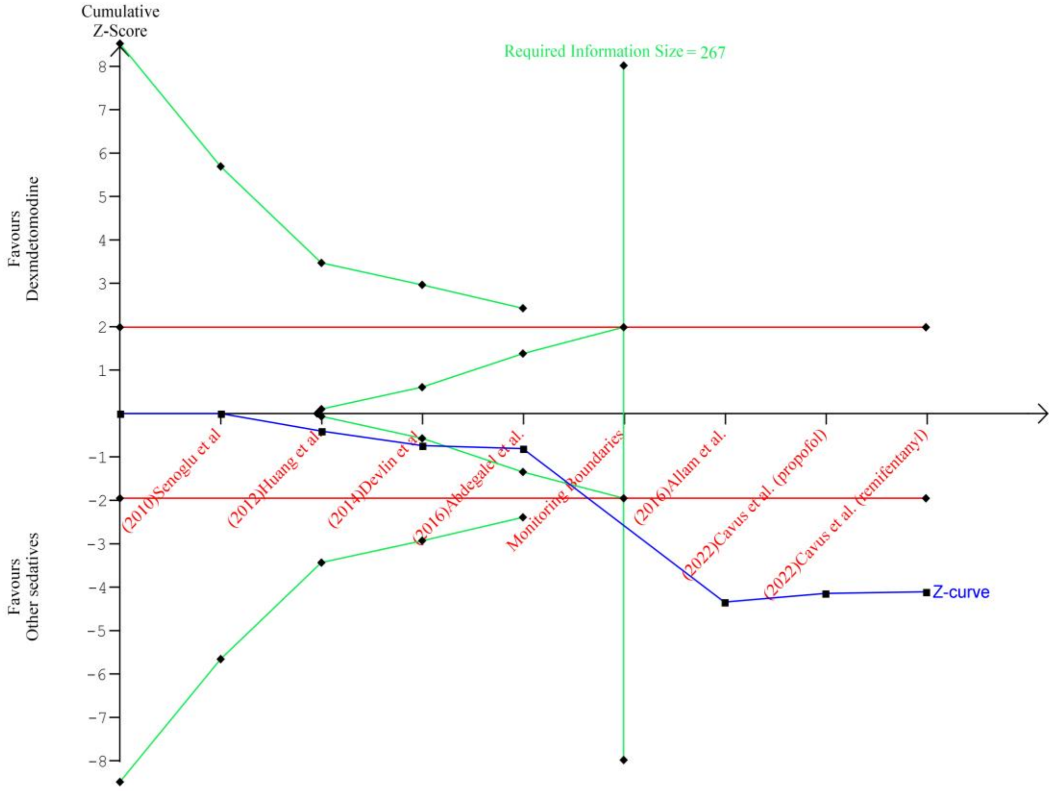

Figure S9. Delirium rates, 28-day mortality, and oversedation events in randomized controlled trials. a-b) delirium and 28-day mortality rates comparisons, respectively, between dexmedetomidine and other sedatives; c-d) delirium and 28-day mortality rates comparisons, respectively, between dexmedetomidine and midazolam; e) oversedation rates comparison between dexmedetomidine and other sedatives.

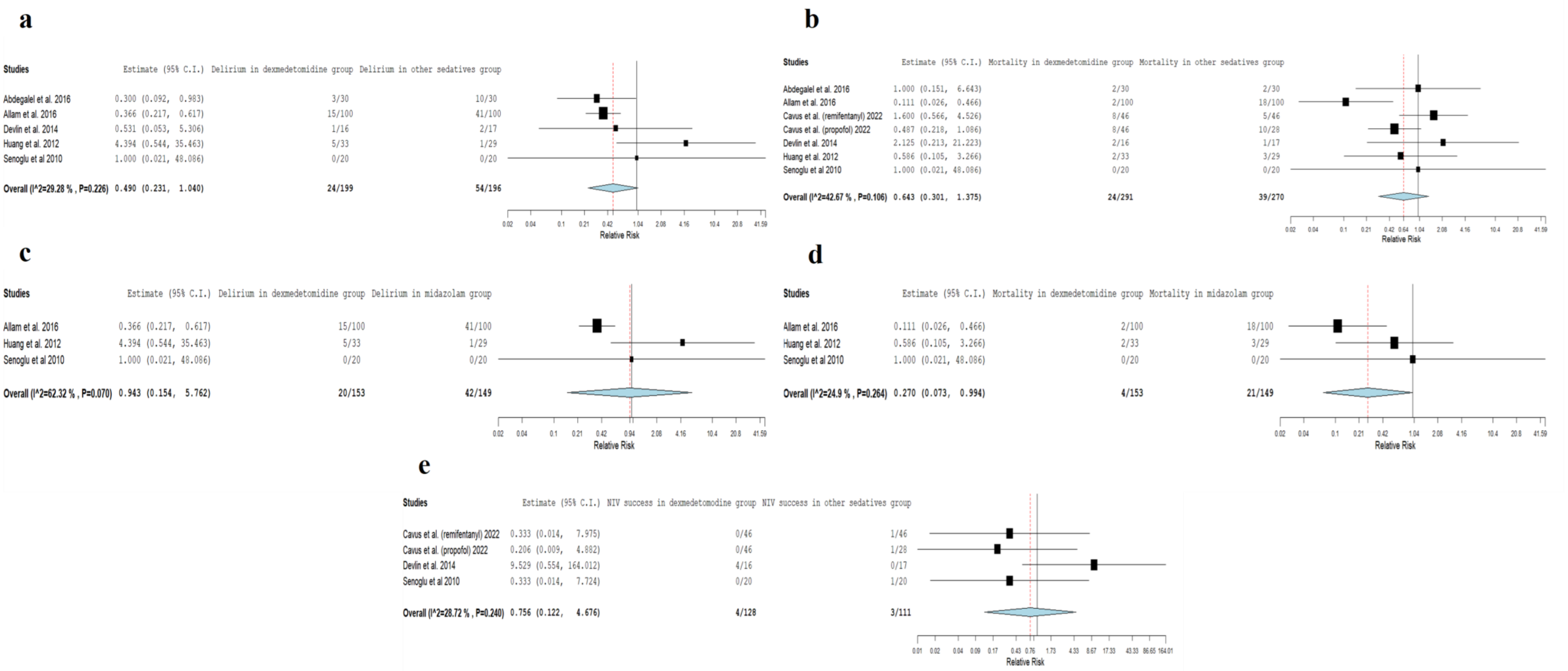

Figure S10. Trial sequential analysis (TSA) of delirium, 28-day mortality, and oversedation rates in randomized controlled trials. a-b) TSA of delirium and 28-day mortality rates comparisons, respectively, between dexmedetomidine and other sedatives; c-d) TSA of delirium and 28-day mortality rates comparisons, respectively, between dexmedetomidine and midazolam; e) TSA of oversedation rate comparison between dexmedetomidine and other sedatives.

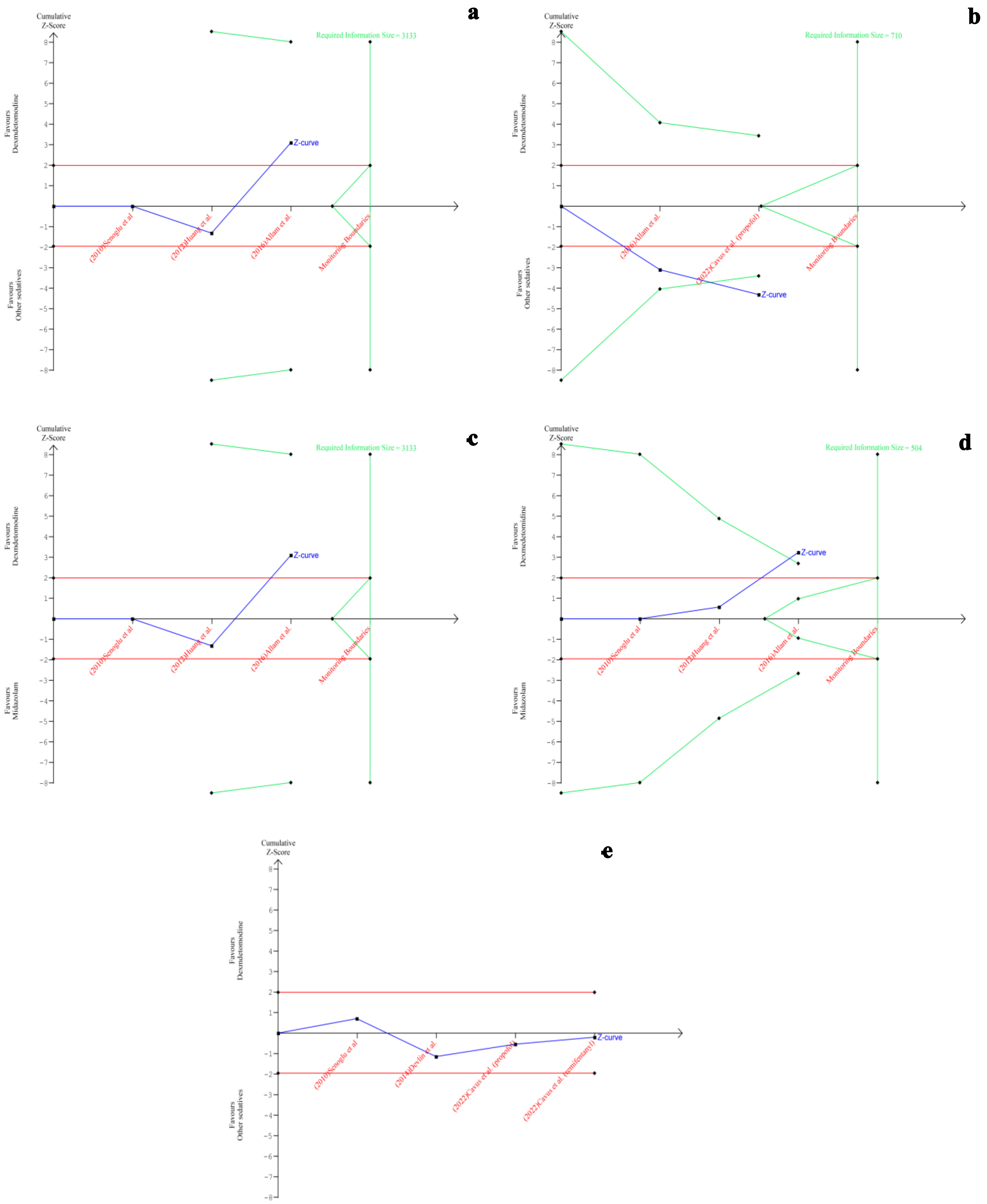

Figure S11. Results from non-randomized clinical trials. a) endotracheal intubation rate (ETI) comparison between dexmedetomidine and other sedatives in retrospective studies; b) 28-day mortality rate comparison between dexmedetomidine and other sedatives in retrospective studies; c) NIV success rate in observational studies; d) ETI rate in observational studies; e) bradycardia events in observational studies; f) hypotension events in observational studies; g) 28-day mortality event in observational studies; h) oversedation rate in observational studies.

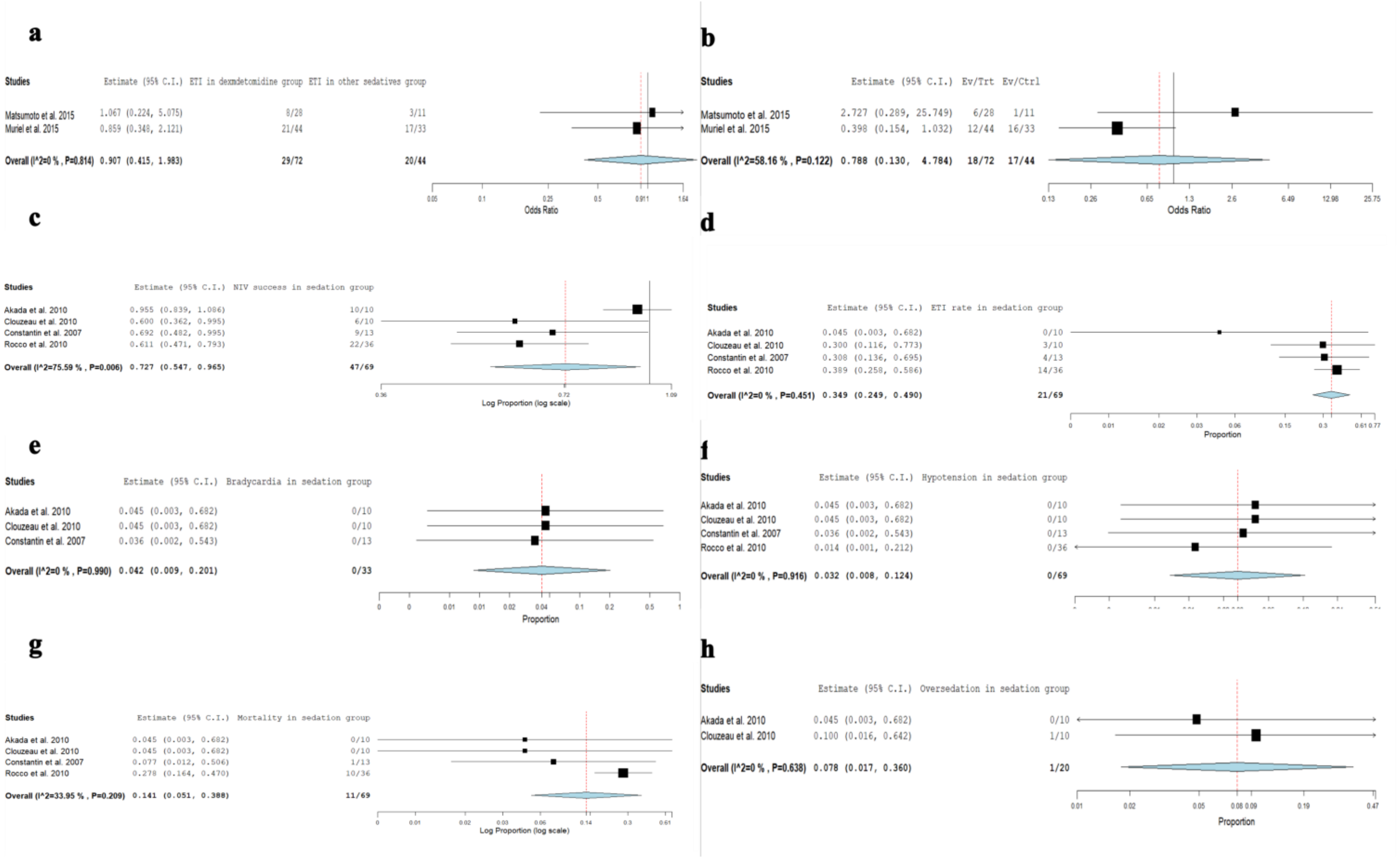

Figure S12. Trial sequential analysis (TSA) of retrospective studies. a) TSA of endotracheal intubation (ETI) rates comparison between dexmedetomidine and other sedatives; b) TSA of 28-day mortality rate comparison between dexmedetomidine and other sedatives.

a

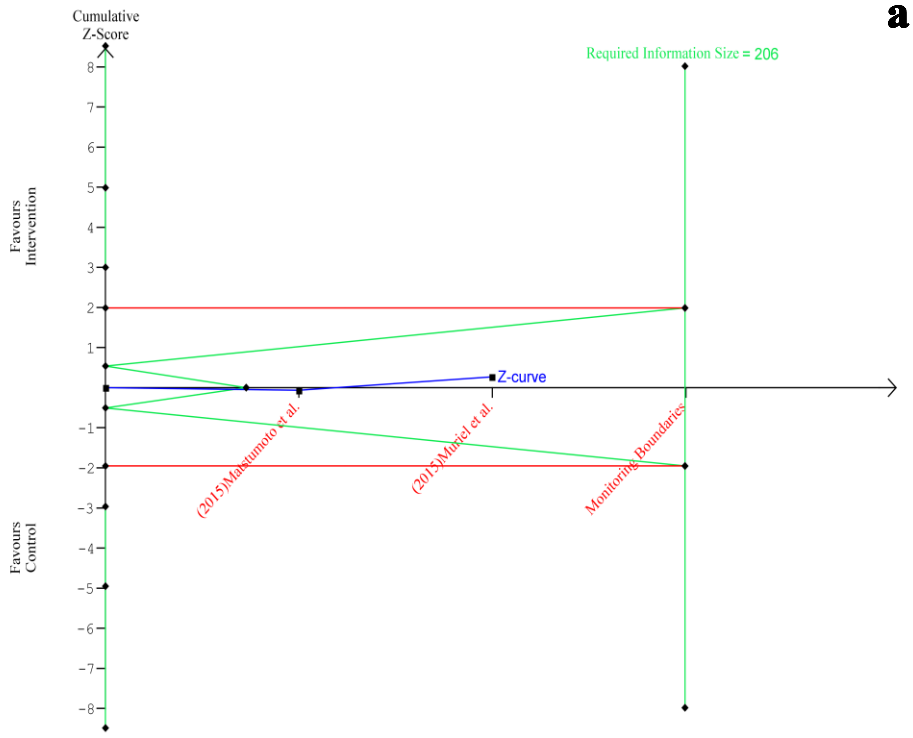

b

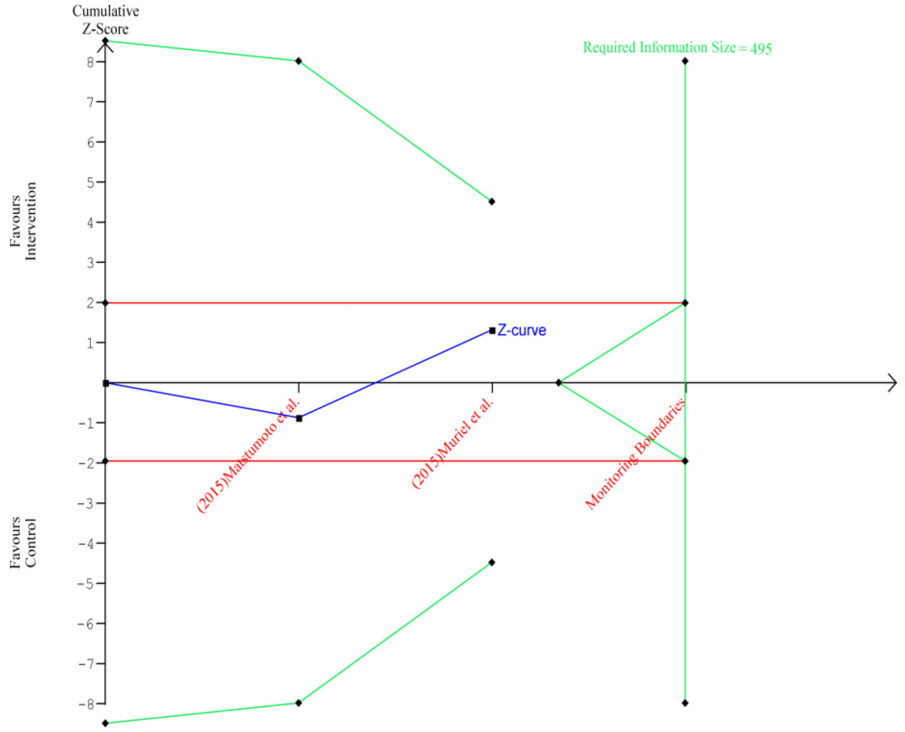

Figure S13. Results from sensitivity analysis of observational studies. a) non-invasive ventilation (NIV) success proportion in sedation group; b) endotracheal intubation (ETI) rate in sedation group; c) bradycardia events in sedation group; d) hypotension events in sedation group; e) 28-day mortality rate in sedation group.

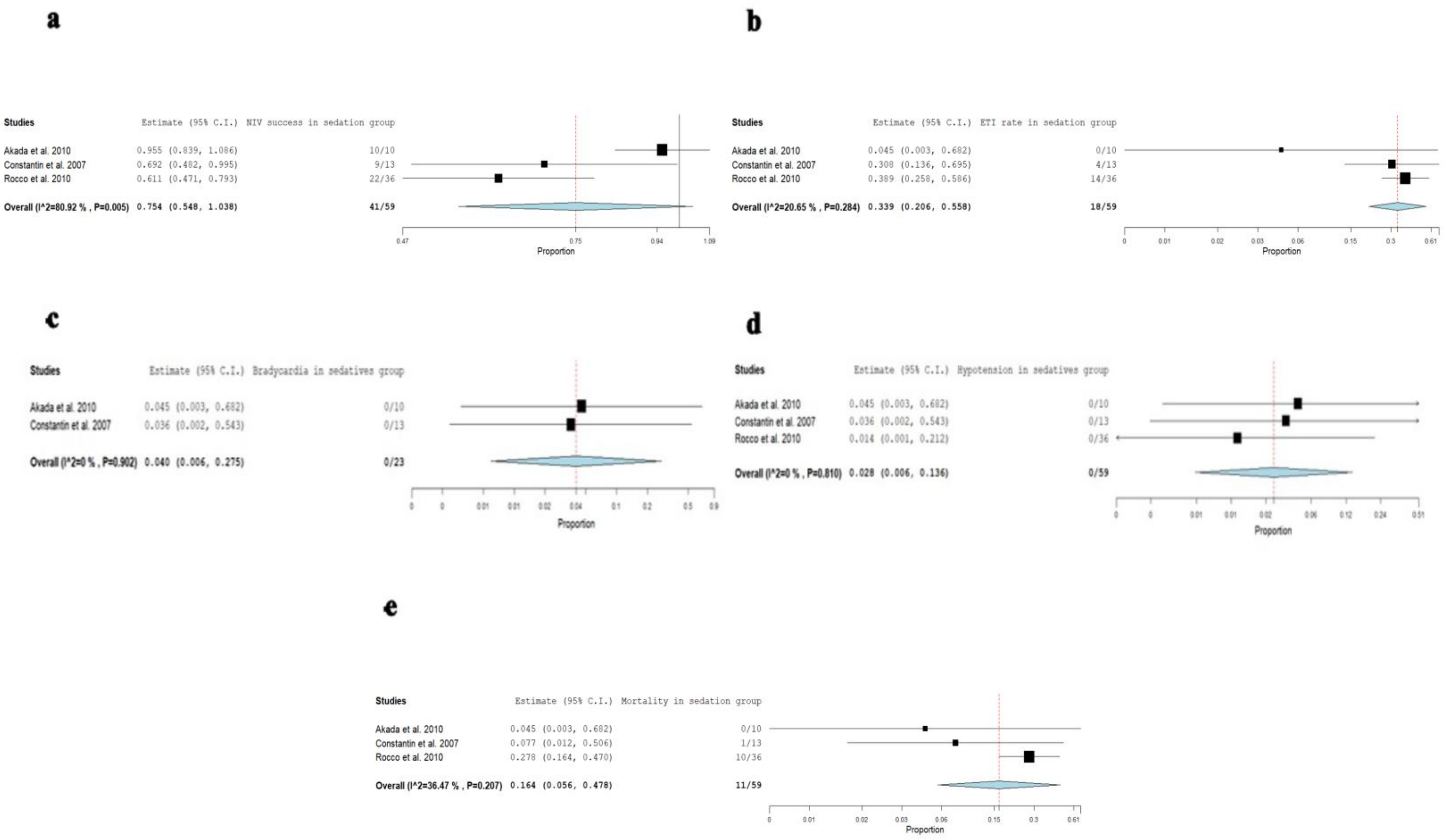

Supplement: Supplementary file 1 [file jpm-16-00385-s001.zip › jpm-4348551-supplementary.pdf]
